# Functional random forest with applications in dose-response predictions

**DOI:** 10.1038/s41598-018-38231-w

**Published:** 2019-02-07

**Authors:** Raziur Rahman, Saugato Rahman Dhruba, Souparno Ghosh, Ranadip Pal

**Affiliations:** 1 Texas Tech University, Department of Electrical and Computer Engineering, Lubbock, Texas 79409 USA; 2 Texas Tech University, Department of Mathematics and Statistics, Lubbock, Texas 79409 USA

## Abstract

Drug sensitivity prediction for individual tumors is a significant challenge in personalized medicine. Current modeling approaches consider prediction of a single metric of the drug response curve such as *AUC* or *IC*_50_. However, the single summary metric of a dose-response curve fails to provide the entire drug sensitivity profile which can be used to design the optimal dose for a patient. In this article, we assess the problem of predicting the complete dose-response curve based on genetic characterizations. We propose an enhancement to the popular ensemble-based Random Forests approach that can directly predict the entire functional profile of a dose-response curve rather than a single summary metric. We design functional regression trees with node costs modified based on dose/response region dependence methodologies and response distribution based approaches. Our results relative to large pharmacological databases such as CCLE and GDSC show a higher accuracy in predicting dose-response curves of the proposed functional framework in contrast to univariate or multivariate Random Forest predicting sensitivities at different dose levels. Furthermore, we also considered the problem of predicting functional responses from functional predictors *i*.*e*., estimating the dose-response curves with a model built on dose-dependent expression data. The superior performance of Functional Random Forest using functional data as compared to existing approaches have been shown using the HMS-LINCS dataset. In summary, Functional Random Forest presents an enhanced predictive modeling framework to predict the entire functional response profile considering both static and functional predictors instead of predicting the summary metrics of the response curves.

## Introduction

Precision medicine plays an important role in the push towards advancing cancer therapy. A significant step in the process involves mapping genetic characterizations to the applied drug sensitivity response. A multitude of approaches have been proposed to address the issue of predictive modeling of drug sensitivity but the results still indicate a significant scope for improvement^1–4^. Crowd-sourced initiatives such as NCI-DREAM conducted Drug Sensitivity Prediction Challenge^2^ enabled the performance evaluation of multiple algorithms on the same dataset while being restricted to smaller number of samples. Recently, a number of pharmacological databases^1,5,6^ have been made public to assist researchers in validating their predictive algorithms using larger biological datasets.

Drug sensitivity information in the form of responses for different doses represented as a curve is becoming more prevalent for cancerous cell lines with the advent of advanced data collection techniques. Such datasets are often referred as *functional* data^7^. Typical approaches for sensitivity prediction predict a summary metric of the entire drug response curve such as *Area Under the Curve* (*AUC*) or *IC*_50_. The problem of predicting a summary metric of the drug response curve has been tackled using a diverse set of regression approaches such as linear regression with regularization, nonlinear regression, kernel based techniques and ensemble based approaches^2,8–10^. Additionally, drug sensitivity prediction modeling has also been proposed based on features extracted using Principal Component Analysis (PCA)^11^.

A primary concern in using a certain drug sensitivity response summary metric is that they fail to describe the entire dose-response effect *i*.*e*., they represent just a particular scenario such as the drug concentration to achieve 50% cell viability (*IC*_50_) or the inflection point of the dose-response fitted curve (*EC*_50_) or the maximal activity reached in the curve (*A*_*max*_)^1^ or the area under the fitted curve (*AUC*). Meanwhile, various functional regression models have been proposed in other research areas to predict the entire response curve^12^. Yu *et al*.^13^ have presented each response curve as a linear combination of known basis functions and grown regression trees using the coefficients of this expansion, while Nerini *et al*.^14^ have proposed functional PCA in the classification method for easy representation of regression trees. The knowledge of the entire drug response curve can answer clinically relevant questions such as what will be the sensitivity at the highest non-toxic dose concentration (toxicity can be estimated using experimentations on normal cells or computational modeling) or the sensitivity at the drug concentration available at the targeted organ (pharmacokinetics estimated using micro-dosing) for that specific patient? Furthermore, a summary metric such as AUC for two different dose response curves might be same even when they might offer different information such as very high sensitivity for high doses for drug A as compared to relatively moderate sensitivity over all drug doses for drug B. Note that drug A at high doses might be better in killing most cancer cells as compared to drug B which will not be apparent through AUC prediction.

Thus, there is a need for entire dose response curve prediction which is not handled directly by existing regression models. In one of our previous works^15^, we have used each dose-response point to build individual regression models for prediction purposes. However, the individual models lack incorporation of the continuous nature of the dose-response curve. In this paper, we are proposing the incorporation of dose-response points or distributions in the generation of regression tree node cost and leaf nodes to improve the accuracy of Random Forest (RF) model for sensitivity prediction. At each regression tree node, region-wise response points or distributions (Gaussian) are considered to calculate the node cost. The leaf nodes store the functional data used to predict the entire dose-response profile for test samples, while the model input consists of genomic characterization in regular form or continuous curve form. We present methodologies that can consider both regular and functional inputs. For analysis purposes, each response curve has been approximated by a linear combination of B-spline functions^13^ and thus, the framework can also be applied in scenarios different from drug sensitivity prediction. We validate our proposed *Functional Random Forest* (FRF) approach using data from the well-known pharmacological databases of Cancer Cell Line Encyclopedia (CCLE)^1^ and Genomics of Drug Sensitivity for Cancer (GDSC)^5^.

The article is organized as follows: The Materials and Methods section compiles the basic steps involved in designing FRF models while discussing the impact of storing functional data in forest leaf nodes and highlighting the region-wise node cost procedures. The Results section provides the performance evaluation of FRF model for both synthetic experiments and actual pharmacological data. Furthermore, it also presents the biological importance of genes selected by FRF. Finally, the Discussion section points out the advantages of using FRF to predict the dose-response curves in the larger context of drug sensitivity prediction and provides possible future research directions.

## Materials and Methods

The idea of Functional Random Forest is based on regular regression tree based Random Forest. Thus, we will first describe the design procedure for regular regression trees and subsequently present the construction of functional regression tree based FRF approach. Before delving into the details of tree construction, we describe the datasets used for this study which will help us establish a number of theoretical assumptions in the methodology.

### Datasets and Preprocessing

For our experiments, we have considered two most comprehensive publicly available cancer pharmacogenomics databases: Cancer Cell Line Encyclopedia (CCLE)^1^ and Genomics of Drug Sensitivity for Cancer (GDSC)^5^. CCLE database was generated by Broad Institute and Novartis Institutes for Biomedical Research. This database includes genetic and pharmacological characterization of 947 human cancer cell lines, together with pharmacological profiling of 24 small molecules (anticancer compounds) across ~500 of these cell lines that encompasses 36 tumor types^1^. The response of a cell line to a specific drug is reported for 7 to 8 dose points ranging from 0.0025 *μM* to 8 *μM*. Additionally, four different drug sensitivity measures *EC*_50_, *IC*_50_, *A*_*max*_ and *AUC* are listed. Note that these measures are features of a dose-response curve fitted from the observed dose-response points. GDSC database was created as part of the Cancer Genome Project^5^ and contains gene expression data for 789 cell lines and drug responses for 714 cell lines. Each cell line has 22,277 probe sets for gene expression yielding a high dimensional feature space. Similar to CCLE, each cell line’s response to the drugs are reported for 7 to 9 dose points where minimum dose ranges from 3 × 10^−5^ *μM* to 15.625 *μM* and maximum dose ranges from 0.008 *μM* to 4000 *μM*. For our experiment, we utilize GDSC *v*5 that lists two drug sensitivity measures *IC*_50_ and *AUC* along with 105 different *IC* values for different levels of cell viability from 0.1% to 100% in each cell line for each drug. Note that these *IC* values are extracted from the complete dose-response curves fitted from the observed dose-response points and extrapolated to 100% cell viability as the curves do not reach 100% at maximum dose for most cell line–drug pairs. Both CCLE and GDSC provide observed dose-response points or fitted curve points which could be utilized as our functional response data. However, the genomic characterization data are available in the stationary format as the expressions are measured before any drug application. Therefore, to demonstrate the functional input and output scenario for our FRF model, we have used data from the Harvard Medical School Library of Integrated Network-Based Cellular Signatures (HMS-LINCS) database, which to our knowledge, is the only publicly available source offering functional responses as well as predictors. HMS-LINCS offers genomic characterization data in the form of Reverse Phase Protein Array (RPPA) expression data for 21 proteins where Phosphorylation state and protein levels were measured in 10 BRAF^*V*600E/D^ melanoma cell lines at 7 different doses and 5 different time points^16^. The cellular response data consists of viability and apoptosis measured in the same cell lines using Fluorescence imaging apoptosis assay for the same 7 doses but 3 different time points^16^. The database contains data for 9 BRAF^*V*600E^ and 1 BRAF^*V*600D^ melanoma cell lines that were exposed to 4 RAF inhibitors and 1 MEK inhibitor at 7 different doses ranging from 3.2 *nM* to 3.2 *μM*. Protein expression data is available for 5 different time points: 1, 5, 10, 24 and 48 hours post drug application and apoptosis data is available for 24, 48 and 72 hours post drug application. For compound sensitivity assessment, two different measures are available: relative viability and mean apoptosis fraction, computed using the number of apoptotic cells and the total number of cells normalized with the DMSO control^16,17^.

**Figure 1:**
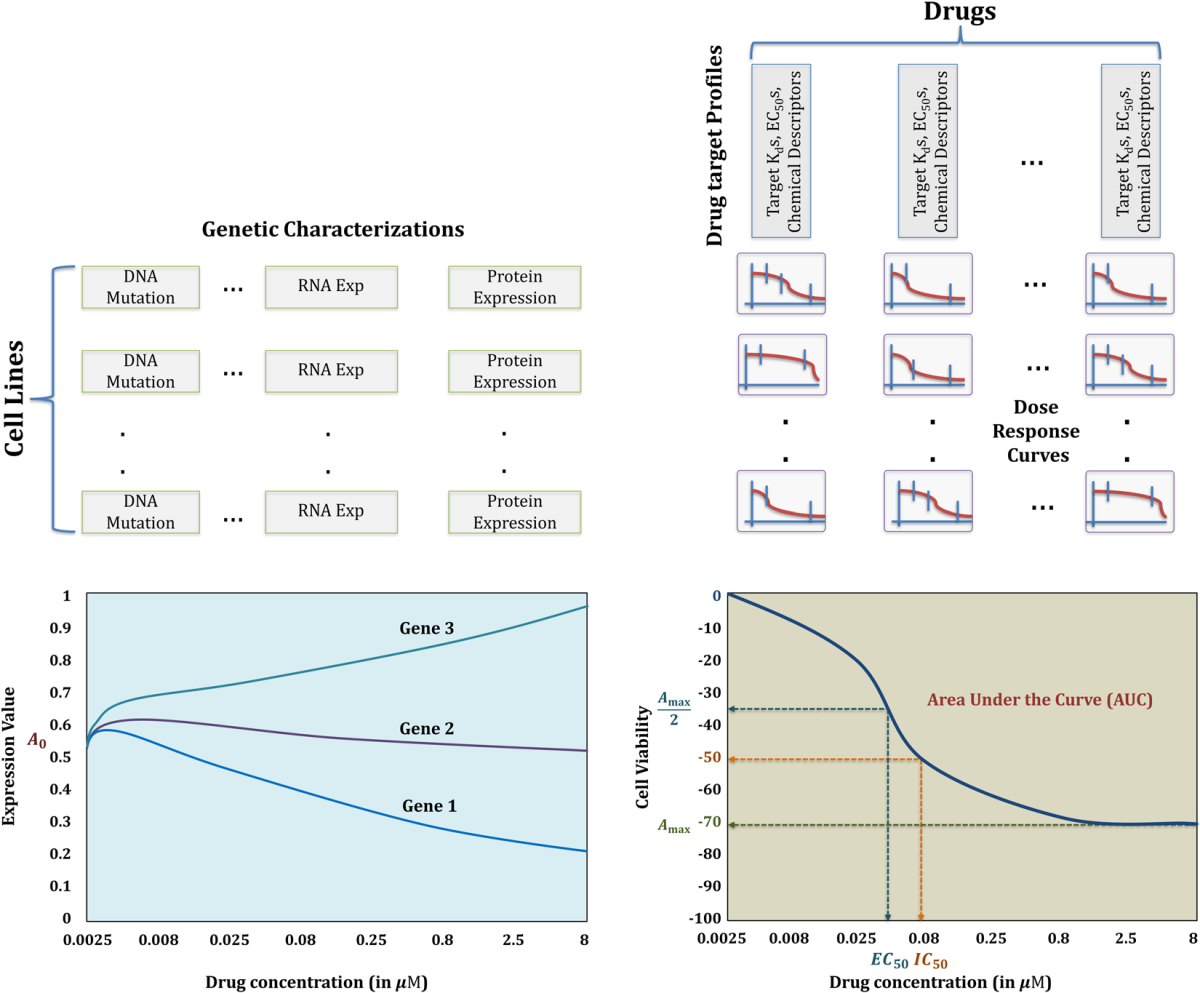
Pictorial representation of the commonly used genomic and functional characterizations.

Figure 1 illustrates the pictorial representations of genomic and functional characterizations data, where the left half shows the static and functional format of genomic characterizations and the right half demonstrates the dose-response curves for various cell line–drug pairs and different summary metrics extracted from such a curve.

### Random Forest Regression

Random Forest consists of a set of *T* un-pruned ensemble of regression trees^18^ that are generated based on bootstrap sampling from the original training data. The bootstrap resampling of the data for training each tree increases the diversity between the trees. Each tree is composed of root node, branch nodes and leaf nodes. For each node of a tree, the optimal node splitting feature is selected from a set of *m* features that are again randomly selected from a feature space of size *M*. If $m\ll M$, the selection of the node splitting feature from a random set of features decreases the correlation between different trees and thus, the average response of multiple regression trees is expected to have lower variance than the individual regression trees. However, there exists a trade-off as a larger *m* can improve the predictive capability of individual trees but also can increase the correlation between trees and void any gains from averaging multiple predictions.

#### Process of splitting a node

Let *x*_*tr*_(*i*, *j*) and *y*(*i*) denote the training input feature *j* and output response, respectively, for sample *i* where $i=1,2,\ldots ,n,\,j=1,2,\ldots ,M$. At any node *η*_*P*_, we aim to select a feature *j*_*s*_ from a random set of *m* (<*M*) features and a threshold *z* to partition the node into two child nodes *η*_*L*_ (left node with samples satisfying ${x}_{tr}(i\in {\eta }_{P},{j}_{s})\le z$) and *η*_*R*_ (right node with samples satisfying ${x}_{tr}(i\in {\eta }_{P},{j}_{s}) > z$). We consider the node cost as sum of square deviances (SSD), *i*.*e*.

1 $$D({\eta }_{P})=\sum _{i\in {\eta }_{P}}\,{(y(i)-\mu ({\eta }_{P}))}^{2}$$

where $\mu ({\eta }_{P})={\mathbb{E}}[y(i\in {\eta }_{P})],\,{\mathbb{E}}[\,\cdot \,]$ denotes the Expected value. Thus, the reduction in cost (*i*.*e*., *reward* function) for partition *γ* at node *η*_*P*_ is given in Eq. (2), where the goal is to select the partition γ* ∈ *η*_*P*_ that maximizes the reward *or*, minimizes the cost.

2 $$\begin{array}{rcl}C(\gamma ,{\eta }_{P}) & = & D({\eta }_{P})-D({\eta }_{L})-D({\eta }_{R})\\ {\gamma }^{\ast } & = & {\rm{\arg }}\mathop{{\rm{\max }}}\limits_{\gamma }\,C(\gamma ,{\eta }_{P})\end{array}$$

Note that for a continuous feature with *n* samples, a total of *n* partitions needs to be checked *i*.*e*., the computational complexity of each node split is *O*(*mn*). During tree generation, a node with *n* ≤ *n*_*size*_ samples is not partitioned any further where *n*_*size*_ is a pre-specified sample size threshold.

Several other approaches have been proposed for tree construction such as applying *Principal Component Analysis* (*PCA*)^19^ in the response matrix^13^. The principal components (PC) not only serve the purpose of dimensionality reduction but is also expected to increase the robustness of the trees. Here, the node cost used to build the trees is given by

3 $$D({\eta }_{P})=\sum _{i\in {\eta }_{P}}\,{(\zeta (i)-\bar{\zeta }(r))}^{T}\,(\zeta (i)-\bar{\zeta }(r))$$

where *ζ*(*i*) denotes a PC based response vector and $\bar{\zeta }(r)$ is the mean vector of PCs^14^. Yu *et al*.^13^ have also considered the use of basis functions to represent the response variables with the node cost written as

4 $$D({\eta }_{P})=\sum _{i\in {\eta }_{P}}\,{({\bf{c}}(i)-{\mu }_{c}({\eta }_{P}))}^{T}\,{\rm{\Phi }}({\bf{c}}(i)-{\mu }_{c}({\eta }_{P}))$$

where **c**(*i*) denotes the vector of basis coefficients, ${\mu }_{c}({\eta }_{P})={\mathbb{E}}[{\bf{c}}(i)]$ and Φ denotes the matrix of basis vector inner products^14^.

#### Forest Prediction

Using the randomized feature selection process, we fit the tree based on *bootstrap* samples $\{({{\bf{X}}}_{1},{Y}_{1}),({{\bf{X}}}_{2},{Y}_{2}),\ldots ,({{\bf{X}}}_{n},{Y}_{n})\}$ from training data. Let us consider the prediction based on a test sample **x** for the tree Θ. Assume that $\tilde{\gamma }({\bf{x}},{\rm{\Theta }})$ be the partition containing **x**, the tree response takes the following form^18,20,21^ with corresponding weights *w*_*i*_(**x**, Θ)

5 $$y({\bf{x}},{\rm{\Theta }})=\sum _{i=1}^{n}\,{w}_{i}({\bf{x}},{\rm{\Theta }})\,y(i)$$

6 $${w}_{i}({\bf{x}},{\rm{\Theta }})=\frac{{{\bf{1}}}_{\{{{\bf{x}}}_{tr}(i)\in \tilde{\gamma }({\bf{x}},{\rm{\Theta }})\}}}{\#\{r:{{\bf{x}}}_{tr}(i)\in \tilde{\gamma }({{\bf{x}}}_{tr}(r),{\rm{\Theta }})\}}$$

Let the *T* trees of RF be denoted by ${{\rm{\Theta }}}_{1},{{\rm{\Theta }}}_{2},\ldots ,{{\rm{\Theta }}}_{T}$ and *w*_*i*_(**x**) to be the average weights over the forest. Then, the average RF prediction for the test sample **x** is given by weighted average of predictions of all *T* trees using the weight vector in (7).

7 $${w}_{i}({\bf{x}})=\frac{1}{T}\,\sum _{j=1}^{T}\,{w}_{i}({\bf{x}},{{\rm{\Theta }}}_{j})$$

8 $$\hat{y}({\bf{x}})=\sum _{i=1}^{n}\,{w}_{i}\,({\bf{x}})\,y(i)$$

### Multivariate Random Forest

Multivariate Random Forest (MRF)^10^ is the extension of the regular RF for joint prediction of multivalued output responses that can be useful in different response scenarios. The primary difference between MRF and the regular RF is in the tree generation step where the node cost is different from $D({\eta }_{P})$ in Eq. (1). In a multivariate output scenario, the difference between a sample point response and the multivariate mean distribution is desirable and can be achieved by using the SSD of the *Mahalanobis distance* measure.

9 $$\begin{array}{rcl}{D}_{MRF}({\eta }_{P}) & = & \sum _{i\in {\eta }_{P}}\,{({\bf{y}}(i)-\mu ({\eta }_{P}))}^{T}\,{{\rm{\Sigma }}}^{-1}\,({\bf{y}}(i)-\mu ({\eta }_{P}))\\ {\rm{where}}\,{\bf{y}}(i) & = & [y(i,1)\,y(i,2)\,\cdots \,y(i,m)]\end{array}$$

where Σ is the covariance matrix, *m* denotes the number of response points, and $\mu ({\eta }_{P})={\mathbb{E}}[{\bf{y}}(i\in {\eta }_{P})]$. The inverse covariance matrix Σ^−1^ is a precision matrix that provides a measure of conditional dependence between multiple random variables. For our analysis, we consider MRF modeling on 8 dose-response points similar to our earlier published study^15^.

### Functional Random Forest

Regular classification and regression trees (CART) work on non-functional variables *e*.*g*., discrete gene expression values and summary metrics shown in Fig. 1. In this section, we consider incorporating functional responses (*e*.*g*., dose-response curves shown in right half of Fig. 1) for building functional random forest (FRF). For this purpose, we have introduced two novel alterations in the regression trees– first, in node cost calculation and second, in regression of the leaf node samples.

#### Node cost calculation

For the construction of regular regression tree based models, partitioning and accuracy measure for each node *η*_*P*_ is achieved using the *deviance criterion* in Eq. (1). However, this criterion only considers a single parameter (*μ*) of the drug sensitivity response while neglecting the shapes of the dose-response curves at each node. To incorporate the shape information of a dose-response curve into the deviance calculation, we propose to *discretize* the entire curve into multiple regions to calculate the node cost in each region separately and then sum the individual deviances to get the total deviance at each node, *i*.*e*.

10 $${\hat{D}}_{FRF}({\eta }_{P})=\sum _{j=1}^{q}\,{\hat{D}}_{r}({r}_{j})$$

where ${\hat{D}}_{r}({r}_{j})$ is the deviance calculated from the *j*^th^ region *r*_*j*_, and *q* is the total number of regions. For the discretization scheme, we choose to discretize the coordinate values as appropriate for the observed data (*e*.*g*., we use the 8 given dose points to divide the dose-response curves into 8 regions in CCLE as compared to GDSC where we utilize the ~100 *IC* response values for discretization). Furthermore, we propose two distinct algorithms for node cost calculation where (i) either the observed dose-response points are used directly or, (ii) the underlying distribution is extracted from these points and various divergence criteria are applied.

#### Node cost calculation using dose-response points

For this approach, we use the observed dose-response data directly and assume the complete curve to be made up of multiple regions each belonging to an observed dose point or response point. Then, the total deviance at each node *η*_*P*_ is measured by calculating the SSD per region^14^ as a measure of ${\hat{D}}_{r}({r}_{j})$ and subsequently using (10).

11 $${\hat{D}}_{r}({r}_{j})=\sum _{i\in {\eta }_{P}}\,\parallel {y}_{j}(i)-{\bar{y}}_{j}{\parallel }^{2}$$

where *y*_*j*_(*i*) denotes the response in region *r*_*j*_ at dose *d*_*j*_ for sample *i*, and ${\bar{y}}_{j}={\mathbb{E}}[{y}_{j}(i\in {\eta }_{P})]$. The criterion described in Eq. (11) considers the region-wise differences rather than the difference in an overall feature of the curve.

#### Node cost calculation using dose-response distributions

In the previous approach, each region consists of ${n}_{P}={\sum }_{i\in {\eta }_{P}}\,i$ response points (*i*.*e*., the number of cell lines examined for the applied drug) at a specific dose *d*_*j*_ and these discrete responses are used to compute the node deviance in (10). However, if a study performs multiple experiments at a certain dose for each individual cell line (*i*.*e*., technical replicates), we can potentially generate a distribution from all the replicates at that specific dose. Therefore, instead of considering a single response value *y*_*j*_(*i*) for cell line *i* at dose *d*_*j*_, we can alternatively calculate the node cost by approximating the response by a probability distribution, *f*_*j*_. The modified splitting criterion for this scenario is given by

12 $${\hat{D}}_{r}({r}_{j})=\sum _{i\in {\eta }_{P}}\,{C}_{f}({{\rm{\Phi }}}_{i},\hat{{\rm{\Phi }}})$$

13 $${\rm{where}}\,{C}_{f}({{\rm{\Phi }}}_{i},\hat{{\rm{\Phi }}})=\sum _{{\rm{\Omega }}}\,\hat{{\rm{\Phi }}}{f}_{j}(\frac{{{\rm{\Phi }}}_{i}}{\hat{{\rm{\Phi }}}})$$

Here, ${C}_{f}(\,\cdot \,,\cdot \,)$ is called the *f-divergence* of the probability distribution, Ω is the distribution range, and $\hat{{\rm{\Phi }}}$ is the mean distribution at node *η*_*P*_ derived using mixture distribution^9^. There are various ways to calculate the *f-divergence* depending on the divergence measure *f*_*j*_(*u*) in Eq. (13). For instance, the *Kullback-Leibler* (*KL*) *divergence*^22^ is obtained with ${f}_{j}(u)=u\,\mathrm{ln}(u)$

14 $${K}_{f}({{\rm{\Phi }}}_{i},\hat{{\rm{\Phi }}})=\sum _{{\rm{\Omega }}}\,{{\rm{\Phi }}}_{i}\,\mathrm{ln}(\frac{{{\rm{\Phi }}}_{i}}{\hat{{\rm{\Phi }}}})$$

And, the *Hellinger Distance*^23^ is generated using ${f}_{j}(u)={(\sqrt{u}-1)}^{2}$

15 $${H}_{f}({{\rm{\Phi }}}_{i},\hat{{\rm{\Phi }}})=\sum _{{\rm{\Omega }}}\,{(\sqrt{{{\rm{\Phi }}}_{i}}-\sqrt{\hat{{\rm{\Phi }}}})}^{2}$$

#### Functional regression using dose-response curves

Regular regression tree response for a new sample is based on averaging the responses in the leaf node reached by the new sample. Since the responses considered in a regular regression tree are individual points, a simple averaging of the values suffices. For our FRF scenario, each leaf node consists of a set of functional responses and therefore, we need to modify the final prediction as described below.

Given that we have dose-response points, we can potentially fit a spline curve through these points to represent the dose-response as a continuous curve. In recent pharmacological studies, the curve fitting normally consists of sigmoidal, linear or constant functions^1^. In our algorithm, we have considered the *generalized B-spline* fitting for the dose-response curves. To perform Functional Random Forest (FRF) prediction using the spline-fitted curves, we store the curve points for each sample in the leaf nodes instead of a specific feature (*i*.*e*., *IC*_50_ or *AUC*). In the prediction step, for a test sample **x**, we consider the training response set ${{\bf{y}}}_{j}={y}_{j}(i\in {\eta }_{P})$ at each dose *d*_*j*_ separately from the stored dose-response curves in node *η*_*P*_ and fit a Gaussian distribution *N*_*j*_. The mode of this distribution (*i*.*e*., peak) indicates the highest response probability for **x** at *d*_*j*_ and we pick the corresponding response value ${\hat{y}}_{j}$ as our final prediction.

16 $$\begin{array}{rcl}{{\bf{y}}}_{j} & \sim  & {N}_{j}({\bf{y}};\,{\mu }_{j},{\sigma }_{j}^{2})\,{\rm{where}}\,{\mu }_{j}\,:\,={\mathbb{E}}[{{\bf{y}}}_{j}],\,{\sigma }_{j}^{2}\,:\,=\mathrm{Var}[{{\bf{y}}}_{j}]\\ {\hat{y}}_{j}({\bf{x}}) & = & {\rm{\arg }}\mathop{{\rm{\max }}}\limits_{y}\,{N}_{j}({\bf{y}};\,{\mu }_{j},{\sigma }_{j}^{2})\end{array}$$

**Figure 2:**
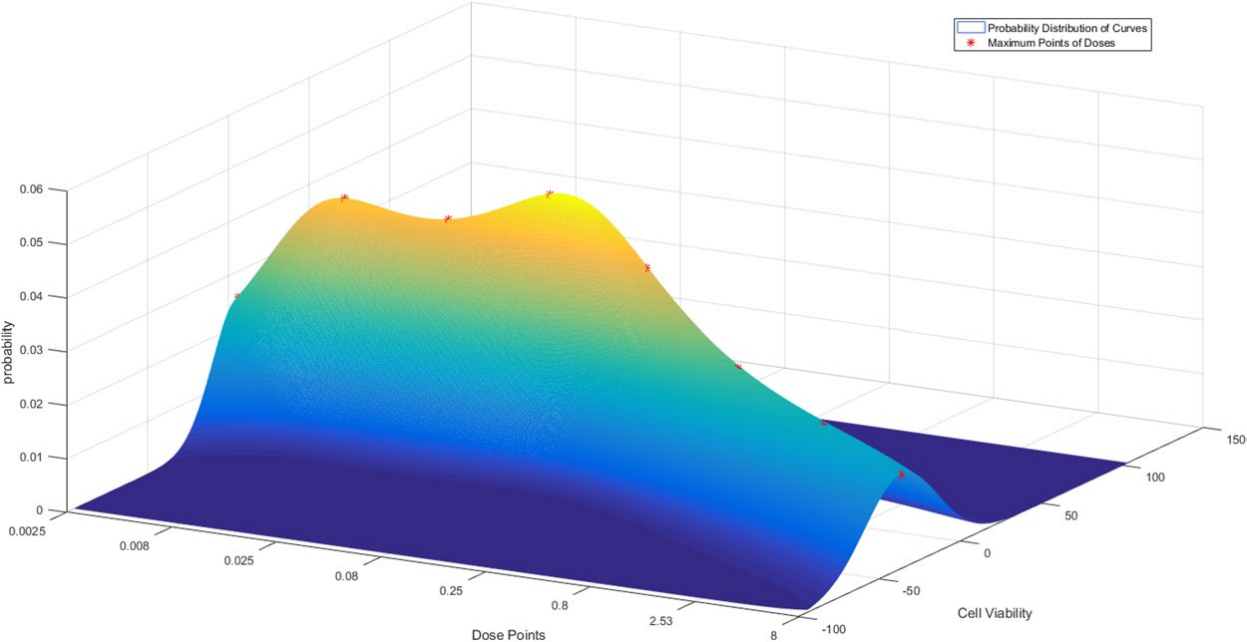
Drug sensitivity probability distributions at a node for Functional Random Forest prediction where the asterisks (*) indicate modes of distributions at 8 dose points ranging from 0.0025 *μM* to 8 *μM*.

The process is then repeated for all dose levels to generate the functional prediction, $\hat{{\bf{y}}}({\bf{x}})$. Figure 2 illustrates a representative case where the different response probability distributions are displayed for multiple dose levels. Here, the asterisks (*) on the 3D surface denote the distribution modes at different doses that are used to perform the functional prediction. Subsequently, we can use this predicted curve to estimate the conventional drug sensitivity measures such as *AUC*, *IC*_50_ and *EC*_50_.

### Function-to-function regression with FRF

Drug sensitivity predictive algorithms normally train regression models on genomic characterizations represented by stationary values such as pre-treatment gene expression (Fig. 1). However, if gene (or protein) expression can be measured post drug application at different doses and/or various time points, the input variables can be modeled as curves representing the dose-expression functions at the corresponding dose points. An example of such functional data is shown in lower left half of Fig. 1 where the functional input-output data is obtained from the HMS-LINCS^16,17^ database. In this section, we consider a scenario where the HMS-LINCS protein expressions following drug administration is available along with the resulting dose-responses in terms of cell viability.

**Figure 3:**
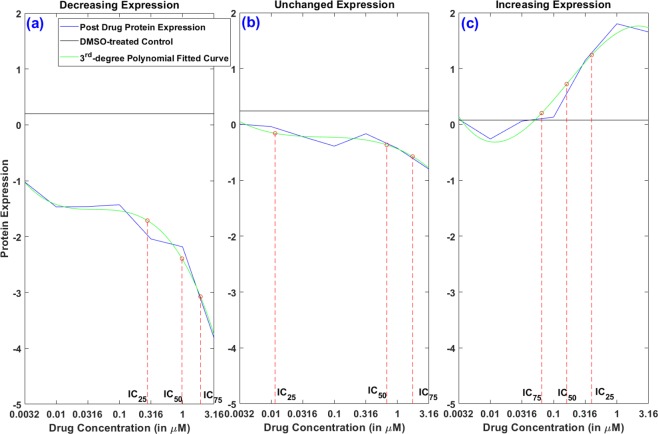
Illustration of obtaining different *IC* values using observed protein expression points and the corresponding 3^*rd*^ degree polynomial fitted curve overlaid after AZ-628 administration in cell line C32 for (**a**) protein *p-S*6 with a decreasing trend, (**b**) protein *p-mTOR* with minor changes, and (**c**) protein *cPARP* with an increasing trend.

Here, we consider a couple of ways to convert the functional data into functional features which are eventually used as model inputs. Similar to the drug sensitivity summary metrics generated from the dose-response curves, we can use the genomic characterization curve to extract features such as *AUC* and *IC*_50_. For calculating *AUC*, a reference line (similar to the zero viability line for drug sensitivity) is required and we utilize the available DMSO-treated control RPPA data^16^ for this purpose. Figure S1 displays a representative dose-expression curve post drug application with the DMSO-treated control line where the shaded area in between is the desired *AUC*. For this representative protein (p-S6), the expression values are decreasing with increases in dose levels which is the most common scenario. However, for a few cases, the protein expressions either remain almost similar or go up as dose increases. For such proteins, we only consider the expression values below our reference DMSO-treated control line (Fig. 3). Along with *AUC*, we also calculate different *IC* values *i*.*e*., *IC*_25_, *IC*_50_ and *IC*_75_ to be considered as predictor features. To arrive at the *IC* values, we perform 3^*rd*^ degree polynomial fitting on the observed protein expression data at different doses and record the different *IC* values using the corresponding percentile points between the lowest and highest expression values (*e*.*g*., *IC*_25_ is the dose where the 25^th^ percentile point is located). Figure 3 illustrates three representative protein expression fitted curves with corresponding *IC*_25_, *IC*_50_ and *IC*_75_ points demonstrating the different behaviors described above *i*.*e*., expression values are either (a) mostly decreasing, (b) almost unchanged, or (c) mostly increasing with dose.

Another way of extracting the functional curve features is to rank the curves according to their slopes (*i*.*e*., rate of change). Furthermore, a curve can be ranked by its position compared to the other curves *i*.*e*., if a curve contains >50% dose points with higher protein expression values compared to another curve, the former will get a higher rank than the later and the process will go on until all curves are ranked.

### Accession codes

Source code for Functional Random Forest is available at: https://github.com/razrahman/Functional-Random-forest.

## Results

In this section, we apply Functional Random Forest modeling on both synthetic and experimental datasets for performance evaluation and comparison analysis with both univariate and Multivariate Random Forest models.

### Application of FRF on synthetic data

**Figure 4:**
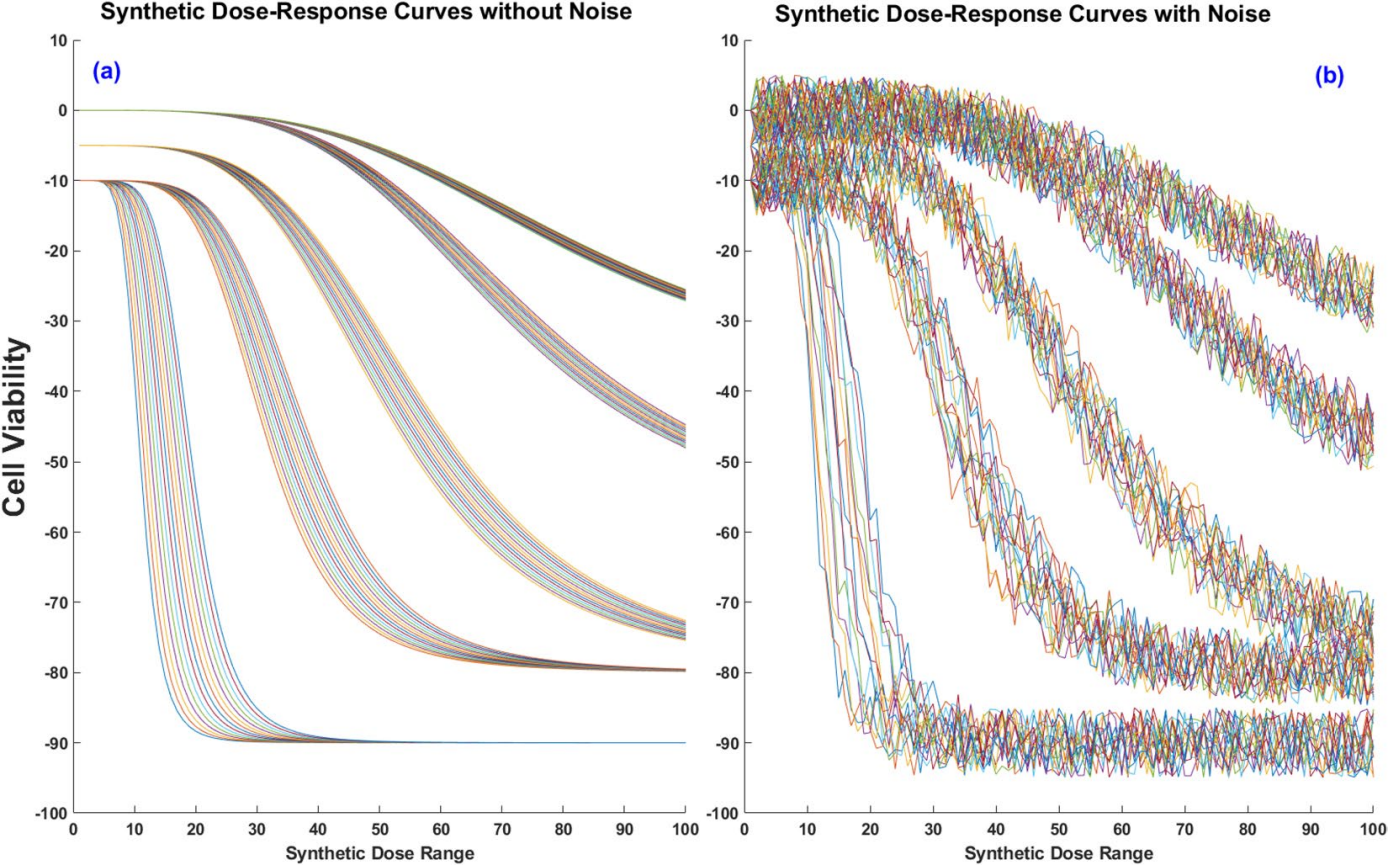
Synthetic dose-response curve examples– (**a**) without noise, (**b**) 5% additive noise.

**Figure 5:**
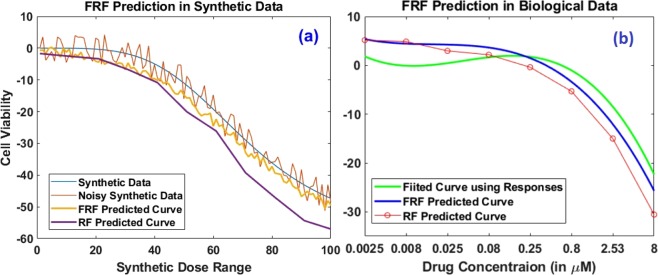
Performance comparison for Functional Random Forest and Random Forest for both synthetic data and CCLE data. (**a**) For noisy synthetic data, FRF can follow the actual response variations even though it was modeled using noisy data while RF fails to follow the trend in higher dose levels, (**b**) For fitted dose-response curve in CCLE Liver cell line SNU449 post Erlotinib administration, FRF prediction again outperforms RF prediction.

We first evaluate the performance of FRF using a synthetic experiment. The design matrix has been generated by extracting 10 different features from five different clusters. Each cluster is derived from a Gaussian distribution and the range of the distribution for each cluster has limited overlap with others. Furthermore, we add 10 additional noise features to increase the correlation between samples from different clusters. Subsequently, we have a design matrix of size 75 × 20 (15 samples each from 5 clusters and 20 covariates with 10 relevant & 10 spurious features). For the output, we create a target matrix of size 75 × 101 where 101 is the number of different synthetic dose levels. The response values are sampled from the 4-parameter sigmoidal model^1^ in Eq. (17) and shown in Fig. 4 for both noiseless and noisy cases, *i*.*e*.

17 $$y(d)={A}_{0}+\frac{{A}_{{\rm{\max }}}-{A}_{0}}{1+{(\frac{I{C}_{50}}{d})}^{\theta }}$$

where *A*_0_, *A*_max_ & *θ* are fixed but *IC*_50_ differs slightly for each curve in a certain cluster while *d* is the applied dose level. We also look into the effect of additive noise in targets as shown in Fig. 4 where (a) displays the target curves *without* noise, and (b) displays the targets with 5% *additive noise*. Table 1 shows the performance of FRF as compared to regular RF for different numbers of trees, folds and noise levels (%). From Table 1, we observe that FRF displays an overall superior performance to RF in all cases, especially improving the model performance by as much as 25% as the noise level increases. A potential reason for this performance boost is the ability of FRF to incorporate the *shape* of the response curves, as shown in Fig. 5(a) where FRF is able to follow a noisy synthetic data curve which RF fails to predict, especially for higher doses.

**Table 1:** Normalized Mean Absolute Errors (NMAE) for prediction of synthetic data dose-responses with varying noise levels using RF and FRF.

#Trees	#Folds	Noiseless		5% Noise		10% Noise		20% Noise	
		RF	FRF	RF	FRF	RF	FRF	RF	FRF
50	5	0.037	**0**.**029**	0.039	**0**.**034**	0.045	**0**.**034**	0.063	**0**.**051**
	10	0.036	**0**.**028**	0.034	**0**.**028**	0.043	**0**.**035**	0.060	**0**.**049**
100	5	0.039	**0**.**030**	0.039	**0**.**034**	0.044	**0**.**036**	0.063	**0**.**049**
	10	0.036	**0**.**029**	0.035	**0**.**030**	0.042	**0**.**035**	0.059	**0**.**047**
150	5	0.041	**0**.**034**	0.036	**0**.**030**	0.047	**0**.**037**	0.060	**0**.**049**
	10	0.031	**0**.**027**	0.034	**0**.**029**	0.042	**0**.**034**	0.060	**0**.**047**
Improvement			24%		17%		25%		25%

The different numbers of folds are used in training & test data separation. Bold values indicate the best performances.

### Application of FRF on biological data

For performance evaluation of Functional Random Forest using actual biological data, we have used three different sources– CCLE, GDSC and HMS-LINCS. The sections below provide the results and corresponding discussion for all three databases.

#### Application on CCLE dataset

CCLE provides cell line sensitivity data with 7 to 8 dose-response points. For our analysis, we consider the cell lines with 8 points only and thus, we have 8 different regions for node cost calculation in Eq. 10. Tables 2 and 3 display the predictive performance of FRF for both node cost calculation algorithms *i*.*e*., using observed dose-response points and underlying distributions. For node cost calculation using distributions, we provide results for both KL divergence and Hellinger distance measures in Eqs (14 and 15). Additionally, we compare the results from the FRF models with standard RF methodology. Tables 2 and 3 provide overall performance comparisons for three different models: (a) regular Random Forest (RF), (b) Functional Random Forest with conventional averaging at the Leaf node (FRFL), and (c) Functional Random Forest with averaging of the dose-response curves at the leaf node (FRF). Note that FRF considers the functional curves for both node cost evaluation *and* response prediction at the leaf nodes, whereas FRFL considers the functional curves for node cost evaluation only and generates the prediction using the conventional means of averaging of a specific summary metric (*e*.*g*., *IC*_50_ or *AUC*) stored at the leaf node. All the results are reported for 5 fold cross-validation with 150 trees in each model along with 10 features for node splitting (*m* = 10) and minimum leaf size of 10. We note that both functional approaches (*i*.*e*., FRFL and FRF) perform better than the regular RF model for all the presented scenarios. We also compare the results with a different set of parameters which also support the previous conclusion that both FRFL and FRF perform better than the RF. Figure 5(b) shows a representative example of both FRF and RF prediction. Note that we are demonstrating a case where the responses are changing gradually for different doses. Although the performances of both FRF and RF were not stellar in general, the FRF prediction still outperforms RF prediction, especially for higher doses.

**Table 2:** Comparison of predictive performance for AUC from three different approaches: RF, FRFL and FRF with two different model constructions using CCLE data.

Drug	Correlation			MAE		
	RF	FRFL	FRF	RF	FRFL	FRF
Model parameters: #Tree = 150, *m* = 10, minimum leaf size = 10						
Erlotinib	0.4408	0.4498	**0**.**4641**	0.0546	0.0541	**0**.**0464**
Nilotinib	0.3886	0.4318	**0**.**4564**	0.0465	0.0464	**0**.**0376**
PD-0325901	0.4716	0.5057	**0**.**5658**	0.1353	**0**.**1335**	0.1377
PLX-4720	0.2957	0.3137	**0**.**4365**	0.0494	0.0487	**0**.**0396**
TAE-684	0.2757	0.3385	**0**.**3743**	0.0728	0.0717	**0**.**0684**
Model parameters: #Tree = 500, *m* = 50, minimum leaf size = 5						
Erlotinib	0.4381	0.4420	**0**.**4701**	0.0563	0.0557	**0**.**0474**
Nilotinib	0.4216	**0**.**4393**	0.4288	0.0470	0.0471	**0**.**0391**
PD-0325901	0.5928	0.5929	**0**.**6381**	0.1287	**0**.**1282**	0.1322
PLX-4720	0.3738	0.4195	**0**.**5352**	0.0492	0.0480	**0**.**0393**
TAE-684	0.3645	0.3888	**0**.**4211**	0.0711	0.0708	**0**.**0679**

For FRFL and FRF, node cost is calculated using 8 dose regions. Bold values indicate the best performances.

**Table 3:** Comparison of predictive performance for AUC from three different approaches: RF, FRFL and FRF using CCLE data.

Drug	Correlation					MAE				
		KL divergence		Hellinger Distance			KL divergence		Hellinger Distance	
	RF	FRFL	FRF	FRFL	FRF	RF	FRFL	FRF	FRFL	FRF
Erlotinib	0.4408	0.4473	0.4620	0.4265	**0**.**4643**	0.0546	0.0544	**0**.**0466**	0.0552	0.0472
Nilotinib	0.3886	0.4263	0.4601	0.4475	**0**.**5009**	0.0465	0.0459	0.0375	0.0457	**0**.**0373**
PD-0325901	0.4716	0.5149	**0**.**5775**	0.4920	0.5633	0.1353	**0**.**1330**	0.1370	0.1352	0.1386
PLX-4720	0.2957	0.3168	0.4308	0.3314	**0**.**4491**	0.0494	0.0489	0.0398	0.0492	**0**.**0397**
TAE-684	0.2757	0.3245	**0**.**3689**	0.2860	0.3337	0.0728	0.0723	**0**.**0688**	0.0730	0.0697

For FRFL and FRF, node cost is calculated using f-divergences (KL divergence or Hellinger distance) of the response distributions at 8 different doses. Bold values indicate the best performances.

Note that Table 3 considers the dose-responses as probability distributions generated based on the mean and standard deviation (SD) of the responses provided by CCLE. We have fitted a Gaussian distribution using the provided mean and SD of responses for each dose point. The mean distribution at a node is calculated using a mixture of Gaussian distribution assumption. Note that the results in both Tables 2 and 3 provide measures for only 5 representative drugs. Table S1 provides the results for all 24 CCLE drugs.

**Figure 6:**
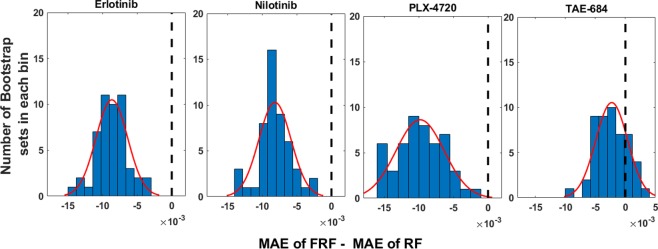
Distributions of MAE differences between FRF and RF predictions for the 50 bootstrap sets using CCLE data.

Both Tables 2 and 3 show the performance measures for 5 fold cross-validation. To demonstrate the robustness of our FRF model compared to RF, we also perform our analysis using bootstrap samples of CCLE data. Considering the total number of samples available for each drug, we extract 50 bootstrap sets of samples to build individual FRF and RF models for each set and then perform sensitivity prediction using the built models. Figure 6 illustrates the distributions of differences between MAE values for FRF and RF model predictions against the number of bootstrap samples for four representative drugs (Fig. S2 provides these distributions for all 24 CCLE drugs). For majority of the sets, MAE of FRF is lower than that of RF yielding negative values in x-coordinate. These distributions clearly demonstrate the superior predictive performance and robustness of FRF as compared to a standard RF. Additionally, Table 4 compares the performance of FRF with that of an MRF model, which also demonstrates the overall superior performance of FRF over MRF for the 8 dose points.

**Table 4:** Comparison of predictive performances of FRF and MRF for 8 different dose points using CCLE data.

Drug	Model	Correlation								
		Dose 1	Dose 2	Dose 3	Dose 4	Dose 5	Dose 6	Dose 7	Dose 8	Mean
Erlotinib	MRF	0.0293	**0**.**2014**	0.1877	0.2901	0.3915	0.4813	0.4942	0.4071	0.3103
	FRF	**0**.**0662**	0.1781	**0**.**2138**	**0**.**3256**	**0**.**4378**	**0**.**4955**	**0**.**5094**	**0**.**4100**	**0**.**3296**
Nilotinib	MRF	−0.0725	**0**.**1966**	0.1550	0.2860	0.3734	0.4255	0.3888	0.1830	0.2420
	FRF	−**0**.**0776**	0.1360	**0**.**2186**	**0**.**3306**	**0**.**4182**	**0**.**4546**	**0**.**4310**	**0**.**2502**	**0**.**2702**
PD-0325901	MRF	0.1402	0.3722	0.4842	0.5395	0.5776	0.5871	0.5668	0.5181	0.4732
	FRF	**0**.**2013**	**0**.**4397**	**0**.**5239**	**0**.**5798**	**0**.**6067**	**0**.**6078**	**0**.**5952**	**0**.**5426**	**0**.**5121**
PLX-4720	MRF	−**0**.**0522**	−0.0137	0.0885	0.1818	0.3986	0.4682	**0**.**5018**	0.3732	0.2433
	FRF	−0.0045	**0**.**1297**	**0**.**1259**	**0**.**2434**	**0**.**4028**	**0**.**4779**	0.4973	**0**.**3772**	**0**.**2812**
TAE-684	MRF	**0**.**1068**	0.1485	0.0045	0.1509	0.3236	0.3448	0.2914	0.2874	0.2072
	FRF	0.0978	**0**.**1615**	**0**.**0541**	**0**.**2358**	**0**.**3654**	**0**.**3867**	**0**.**3736**	**0**.**3008**	**0**.**2470**

All the models are built using 150 trees, *m* = 10 node splitting features and minimum leaf size of 10.

#### Application on GDSC dataset

To demonstrate the versatility of FRF model performance as compared to a traditional RF model, we performed the predictive analysis using another publicly available larger database GDSC. Instead of dose-response points, GDSC v5 provides 105 different *IC* points for dose-response values, extracted from response curves fitted with sigmoidal functions^5^ and extrapolated to reach 100% cellular viability. This extrapolation causes the dose values for *IC*_90_ or *IC*_100_ to be very high and therefore, we consider only the *IC* values indicating ≤80% viability in our models. We design a single FRF model to predict the complete dose-response curve from *IC*_1_ to *IC*_80_ and thereafter, the *AUC*. However, RF is unable to replicate this procedure and therefore, we design 8 separate models to predict 8 different *IC* values in an interval of 10 (*i*.*e*., $I{C}_{10},I{C}_{20},\ldots ,I{C}_{80}$) and one additional model to predict the *AUC*. Table 5 provides the MAE values measured at the 8/*IC* points and *AUC* for both FRF and RF for 5 representative drugs (Table S2 provides the performance comparison for all 140 GDSC (v5) drugs). For all 5 drugs, FRF displays a superior performance in predicting different *IC* and *AUC* values as compared to RF. These results demonstrate the higher efficacy of FRF in the larger context of drug sensitivity prediction for various dose or response points.

**Table 5:** Comparison of predictive performance on GDSC dataset for multiple drug sensitivity measures (*AUC* and 8 *IC* values) using both RF and FRF.

Drug	Model	MAE									
		*AUC*	*IC*_10_	*IC*_20_	*IC*_30_	*IC*_40_	*IC*_50_	*IC*_60_	*IC*_70_	*IC*_80_	Mean
Erlotinib	RF	0.0596	2.0831	1.7472	1.5039	1.3291	1.1948	1.0692	1.0133	1.0304	1.3714
	FRF	**0**.**0486**	**1**.**9813**	**1**.**6597**	**1**.**4382**	**1**.**2694**	**1**.**1357**	**1**.**0361**	**0**.**9867**	**1**.**0095**	**1**.**3146**
Rapamycin	RF	0.0640	**4**.**3771**	3.4771	2.9370	2.5294	2.2000	2.0355	2.0207	2.5359	2.7641
	FRF	**0**.**0636**	4.3905	**3**.**4525**	**2**.**8895**	**2**.**4642**	**2**.**1379**	**1**.**9446**	**2**.**0046**	**2**.**4707**	**2**.**7193**
Sunitinib	RF	0.0963	1.5494	1.5297	1.5542	1.6105	1.6518	1.7013	1.7750	1.8728	1.6556
	FRF	**0**.**0902**	**1**.**5306**	**1**.**5119**	**1**.**5378**	**1**.**5750**	**1**.**6276**	**1**.**6812**	**1**.**7428**	**1**.**8372**	**1**.**6305**
PHA-665752	RF	0.0370	1.4403	1.2665	1.1492	1.0658	1.0002	0.9555	0.9539	0.9485	1.0975
	FRF	**0**.**0259**	**1**.**3522**	**1**.**2051**	**1**.**0999**	**1**.**0149**	**0**.**9546**	**0**.**9054**	**0**.**8954**	**0**.**9097**	**1**.**0422**
MG-132	RF	0.1246	1.6207	1.6688	1.7445	1.7830	1.8549	1.9289	2.0313	2.1509	1.8479
	FRF	**0**.**1070**	**1**.**6062**	**1**.**6479**	**1**.**6968**	**1**.**7541**	**1**.**8117**	**1**.**8794**	**1**.**9619**	**2**.**0857**	**1**.**8055**

For FRF, node cost is calculated using 8 different *IC* regions. Bold values indicate the best performance.

**Figure 7:**
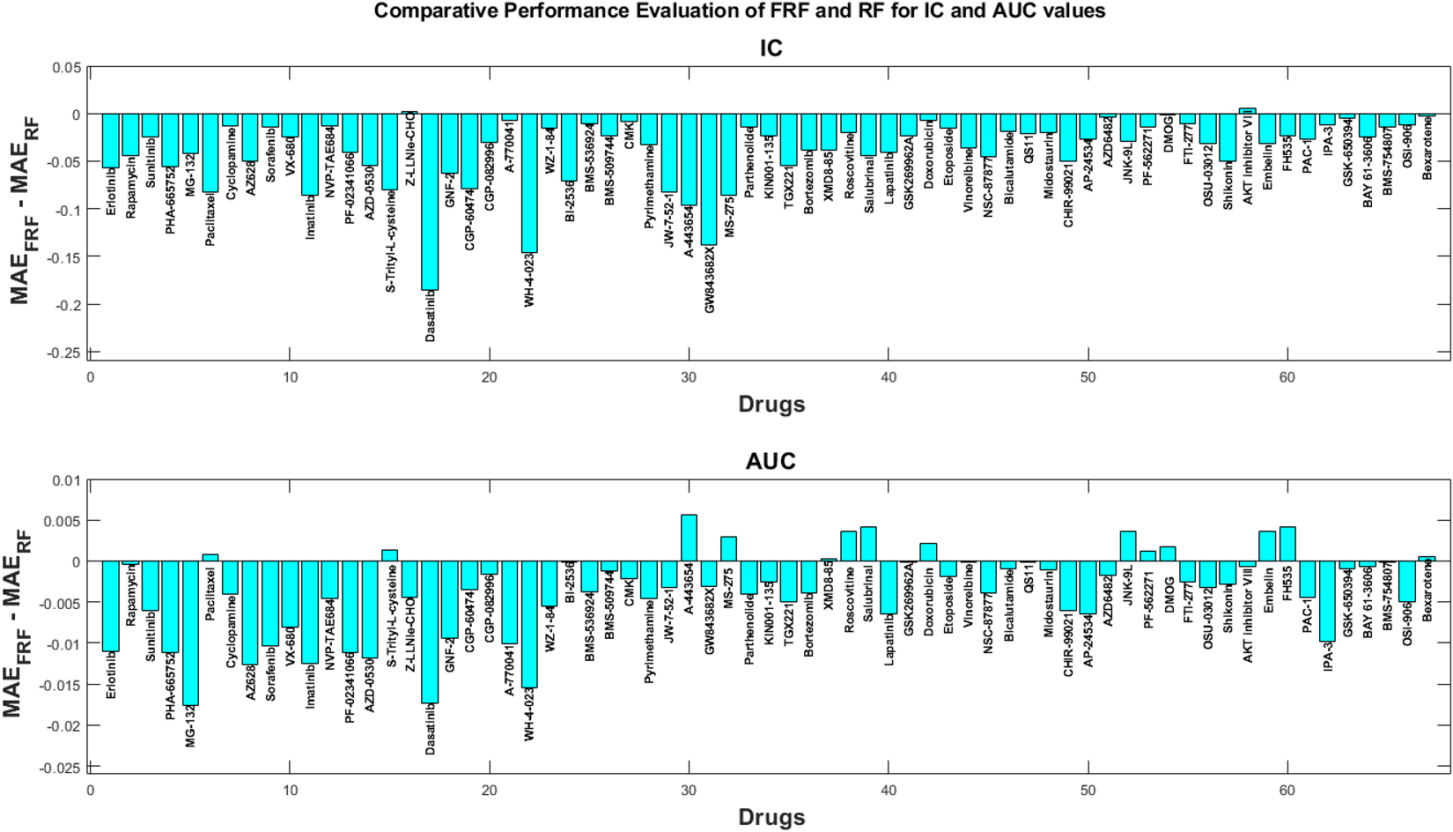
Difference between MAEs of FRF and RF for (i) Mean *IC* values, and (ii) *AUC* values for 70 drugs from GDSC.

Figure 7 illustrates the difference between MAE values of FRF and RF predictions for Mean *IC* and *AUC* values for 70 drugs from GDSC. For mean *IC*, FRF shows superior performance in 68 out of 70 applied drugs, while FRF outperforms RF in 58 out of 70 applied drugs for *AUC* prediction. These results support the conclusion achieved from CCLE data analysis that FRF provides higher predictive accuracy than a regular RF. Figure S3 provides the performance comparison of the rest of the 140 GDSC (v5) drugs.

#### Function-to-function regression using HMS-LINCS

**Figure 8:**
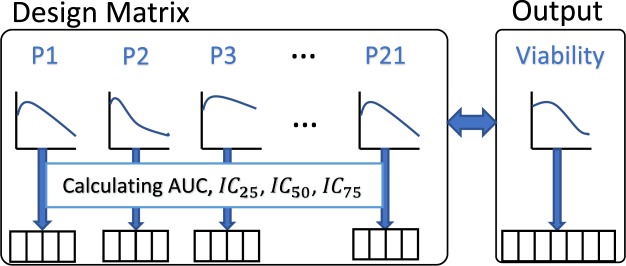
Data extraction procedure for Functional Regression Tree model. From each of the 21 observed protein expression curves, we calculate the *AUC*, *IC*_25_, *IC*_50_ and *IC*_75_ values resulting in a complete feature matrix of 21 × 4. For response modeling, the entire cellular viability curve post drug application is used directly.

As described earlier, the HMS-LINCS database provides functional data for input proteomic expressions (for 21 proteins) and output cellular viability^16,17^ post application of 5 different drugs at 7 different doses in 10 melanoma cell lines at multiple time points. For our analysis, we only use the 48-hour data since it contains complete records for both input and output. Thus, we have 50 samples in total with 143 predictors (*i*.*e*., 21 × 7 − 4 = 143, since we exclude 4 proteins due to missing values). The detailed description of the data extraction framework is provided in section Function-to-function regression with FRF with a pictorial representation in Fig. 8. For our function-to-function regression using FRF, we either consider the 143 predictors directly as input features, or extract the 3^rd^ degree polynomial-fitted dose-expression curve features to use as predictors. As the curve features, we estimate 3 different *IC* points at *IC*_25_, *IC*_50_ and *IC*_75_ and the overall *AUC*, as shown in Figs 3 & S1 for all 21 proteins. Table 6 displays the function-to-function regression results for 3 different input scenarios using FRF. We compare these performances with the performances of dose-wise standard RF models using the 143 expression values as input features for the 50 samples. From Table 6, we observe that FRF provides superior performance as compared to RF for all 3 scenarios while the usage of curve *IC* features provides the highest reduction (~20%) in prediction error. These results clearly demonstrate the potential of FRF in enhancing the predictive modeling performance *via* utilizing the functional input curve features.

**Table 6:** Comparison of predictive performance of RF and FRF with functional data input from HMS-LINCS where *AUC*, *IC*_25_, *IC*_50_ & *IC*_75_ values of proteomic dose-expression curves are used as input features.

Model	Input Feature Description	#Features	#Models	MAE
RF	Protein Expression	143	7	0.2656
FRF	Protein Expression	143	1	0.2602
	*AUC*, *IC*_25_, *IC*_50_ & *IC*_75_ of dose-expression curve	84	1	0.2255
	*IC*_25_, *IC*_50_ & *IC*_75_ of dose-expression curve	63	1	**0**.**2154**

Bold value indicates the best performance.

### Biological validation of the models

A potential model validation approach is to consider the *variable importance measure* (VIM) of the genes. We expect that a better model will have higher feature scores for the significant genes, and thus, in turn will result in a higher biological relevance. Typically in RF based models, VIM (or feature score) is calculated from either the frequency of feature selection, out of bag errors, or permutation measures^24,25^. In this section, we use the frequency based approach to calculate the VIM score from the number of times a gene is considered and the number of times it actually gets selected in splitting the nodes.

18 $${{\rm{VIM}}}_{j}=\frac{\#{\rm{times}}\,{\rm{gene}}\,j\,{\rm{is}}\,{\rm{selected}}}{\#{\rm{times}}\,{\rm{gene}}\,j\,{\rm{is}}\,{\rm{considered}}}=\frac{{m}_{j}^{{\rm{selected}}}}{{m}_{j}^{{\rm{picked}}}}$$

For our FRF models, we have selected the parameters values as #Trees = 500, *m* = 50, minimum leaf size = 5 for a 5 fold cross-validation of CCLE data. Based on these values, all 18,405 CCLE genes gets picked around 600 to 900 times, giving each a fair chance to contribute to the model. The top features of the models (*i*.*e*., genes with higher VIM scores) are then biologically validated in terms of protein-protein interaction (PPI) network enrichment analysis.

There are a number of Bioinformatics resources (*e*.*g*., STRING^26^, GeneMANIA, DAVID etc.) available for evaluation of the number of observed PPIs in a set of selected genes. These interactions have been determined using prior knowledge and information from various interaction sources such as literature text-mining, experiment results, genomic/proteomic databases, gene co-expressions, gene neighborhood, gene fusion and co-occurrences. For CCLE, we have used Affymetrix HG-U133A mapping to convert the top features into corresponding genes. These genes are then provided as the inputs in the STRING database (http://string-db.org/) to extract the known PPI network. Table 7 shows the PPI analysis results for entire genome with a minimum interaction score of 0.15 for the 5 previously considered drugs for both FRF and equivalent RF models. We observe a higher level of connectivity enrichment for the top 200 FRF features as compared to the top 200 RF features in terms of PPI enrichment *p*-value and the ratio of observed to expected number of edges^27^, resulting from possibly the functional collaborations between the products of the FRF genes.

**Table 7:** Protein-protein interaction enrichment analysis for top 200 genes picked from RF and FRF using the whole genome statistical background with a minimum interaction score of 0.15.

Drug	Model	#Nodes	#Edges	Expected #edges	Ratio of observed to expected #edges	PPI enrichment *p*-value
Erlotinib	RF	107	132	127	1.04	0.356
	FRF	105	170	142	**1**.**20**	**0**.**013**
Nilotinib	RF	102	185	162	1.14	0.044
	FRF	101	173	144	**1**.**20**	**0**.**010**
PD-0325901	RF	107	191	187	1.02	0.407
	FRF	113	153	139	**1**.**10**	**0**.**134**
PLX-4720	RF	106	159	147	1.08	0.164
	FRF	111	217	187	**1**.**16**	**0**.**018**
TAE-684	RF	103	159	141	1.13	0.078
	FRF	106	180	151	**1**.**19**	**0**.**011**

## Discussion

**Figure 9:**
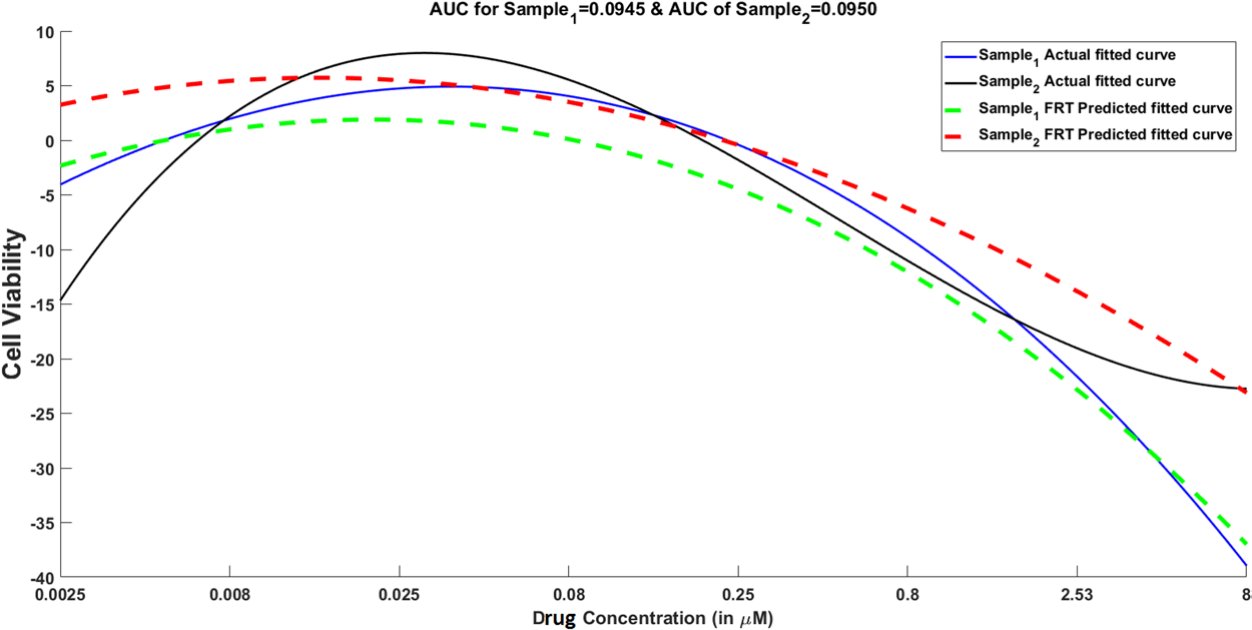
Illustration of different dose-response curves for two cell lines in CCLE post AZD-6244 application with similar AUC values but different responses at higher doses. The complete dose-response profile prediction using Functional Random Forest is able to capture the difference in response behaviors for majority of the doses.

In this article, we have presented an enhancement to Random Forest modeling that can incorporate both stationary and functional inputs to predict functional output. The ability to predict the complete functional dose-response profile can be instrumental in various scenarios. For instance, there can be multiple dose-response curves with similar values of the extracted features (*i*.*e*., *AUC* or *IC*_50_) but they can significantly differ in cytotoxicity or cell viability rate at higher doses. Figure 9 shows an example of this phenomenon where two different dose-response curves for two distinct cell lines in CCLE after AZD-6244 administration have almost the same *AUC* values (*AUC*_1_ = 0.0945, *AUC*_2_ = 0.095) but different rates of cell viability change at doses ≥0.25 *μM*. Figure 9 also demonstrates that FRF is capable of capturing the different response curve behaviors for the two cell lines.

**Figure 10:**
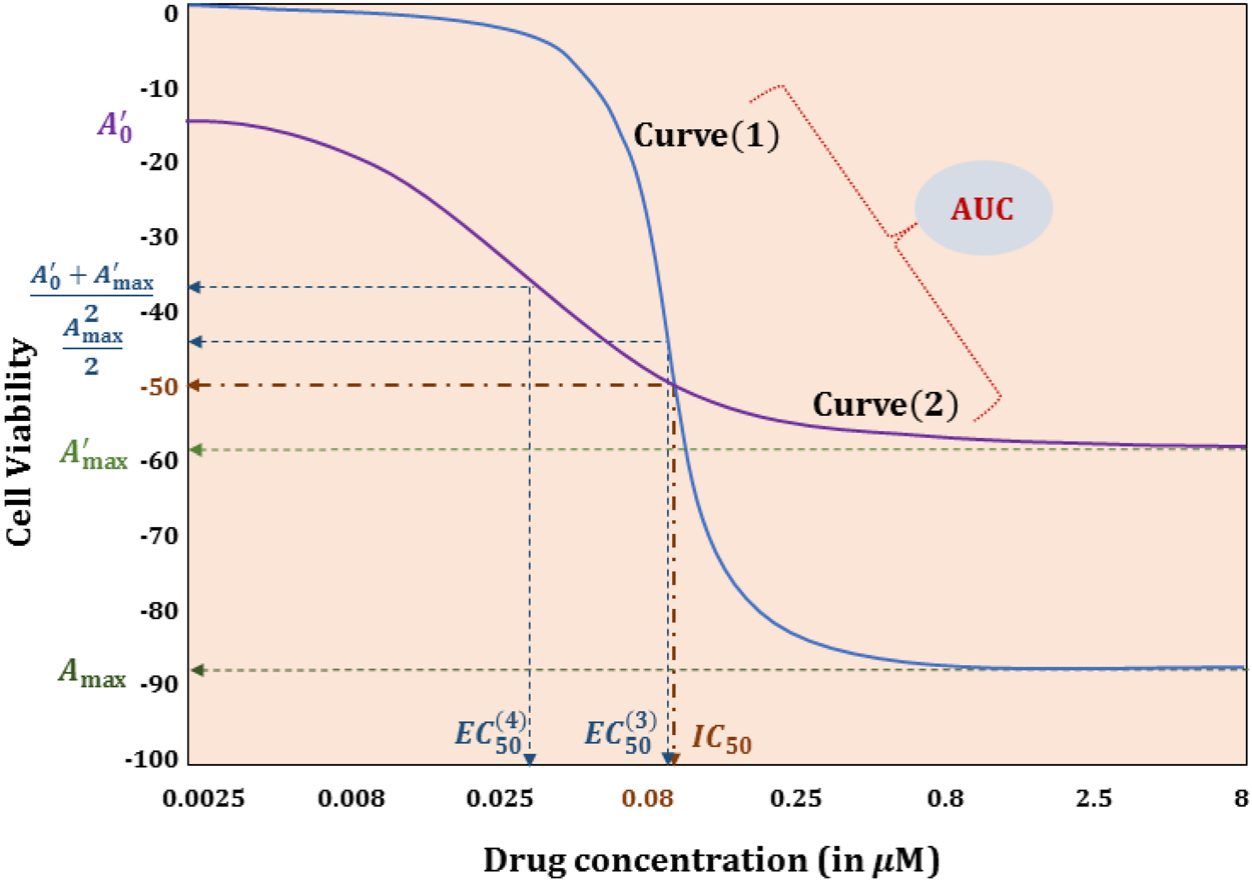
Two different dose-response curves with the same *IC*_50_ and *AUC* values.

Through the application on both synthetic and actual biological data, we have established the superior performance of FRF in predicting dose-response curve summary metrics such as *AUC* and *IC*_50_ as compared to naïve Random Forest model trained on these metrics as output. Furthermore, FRF predicts the entire dose-response profile incorporating the continuous nature of the curve that separate RF models for individual doses fails to capture. We have illustrated this behavior for GDSC dataset by modeling 8 *IC* points using 8 different RFs to generate the dose-response profile which has an inferior performance compared to the continuous curve prediction from FRF (Table 5). Moreover, a major advantage of predicting a complete curve is the visualization of the changes in response across different doses. Figure 10 shows two representative cases of Curve^(1)^ and Curve^(2)^ that has same *IC*_50_ values and similar *AUC* values but their dose-response profiles are significantly different. For instance, a small dose increase above *IC*_50_ will produce significantly higher sensitivity for Curve^(1)^ whereas Curve^(2)^ will have minimal change for dose increases above the *IC*_50_ value. This behavior will not be captured if we only predict the *AUC* or *IC*_50_ summary metric as both the curves have similar *IC*_50_ and *AUC* values. This example illustrates the need for complete dose-response profile prediction in the larger context of drug sensitivity prediction.

There are a number of adjustable parameters available in any regression tree based model (*i*.*e*., minimum leaf size, maximum features used for split, and number of trees in the forest) that we can change to get optimal performance, as illustrated in Table 2. Note that increasing the model complexity has similar impact on both RF and FRF models with FRF retaining its superior performance over RF but with a higher computational demand. However, we also observed several drugs in CCLE (e.g., 17-AAG, AZD-6244, Paclitaxel, PD-0325901) for which the prediction errors (MAE) for both FRF and RF are quite high. For these drugs, the dose-response points at different doses for the available cell lines are stretched out and the resulting fitted curves or summary metrics show significant variations which cannot be captured by any Random Forest based model since it employs an smoothing strategy (averaging) in the leaf nodes to provide estimates around the mean prediction. We are currently looking at different types of regression modeling to solve this issue of bias in prediction. We also hope to further extend this work *via* the incorporation of joint prediction of multiple correlated dose-response profiles while preserving the output dependency structure.

## Supplementary information

Supplementary: Functional Random Forest with applications in dose response predictions
