# Supplementary material for: Functional random forest with applications in dose-response predictions
Source: Sci Rep. 2019 Feb 7;9:1628. doi: 10.1038/s41598-018-38231-w (PMC6367407; doi:10.1038/s41598-018-38231-w)
Supplement: Supplementary file 1 — Supplementary: Functional Random Forest with applications in dose response predictions [file 41598_2018_38231_MOESM1_ESM.pdf]

Supplementary: Functional Random Forest with applications in dose response predictions

Raziur Rahman, Saugato Rahman Dhruba, Souparno Ghosh and Ranadip Pal

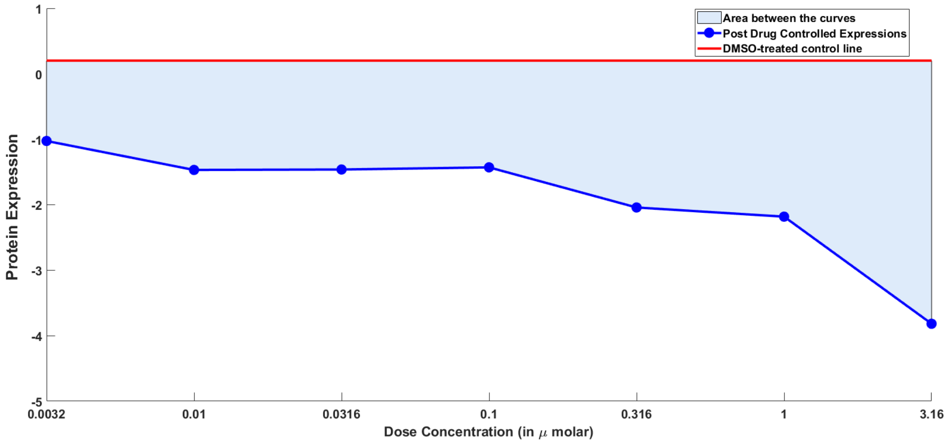

Figure S1: Post Drug and DMSO-treated Protein Expression for Drug AZ-628 for Cell Line C32 for p-S6 protein

Table S1: Correlation coefficient and Mean Absolute Error (MAE) between actual and predicted drug sensitivities (AUC) for 3 methods; RF, FRFL and FRF with three different model constructions. In FRFL and FRF, node cost is calculated using 8 dose regions. Here, results of all 24 drugs of CCLE database are shown.

Drug Name	FRF node cost criteria	Correlation Coefficient			Mean Absolute Error		
		RF	FRFL	FRF	RF	FRFL	FRF
17-AAG	Points from 8 dose regions	0.3181	0.338	**0.4213**	0.1009	**0.1002**	0.1108
	Distributions from 8 dose regions (KL divergence)		0.234	0.3317		0.1022	0.1146
	Distributions from 8 dose regions (Hellinger Distance)		0.2962	0.3424		0.1018	0.1144
AZD-0530	Points from 8 dose regions	0.3095	**0.3459**	0.3041	0.0684	0.0676	**0.0618**
	Distributions from 8 dose regions (KL divergence)		0.3127	0.2997		0.0689	0.0622
	Distributions from 8 dose regions (Hellinger Distance)		0.3023	0.2926		0.0692	0.0622
AZD-6244	Points from 8 dose regions	0.4866	0.5242	**0.5716**	0.1009	0.0994	**0.0994**
	Distributions from 8 dose regions (KL divergence)		0.4809	0.5296		0.1015	0.1014
	Distributions from 8 dose regions (Hellinger Distance)		0.4889	0.522		0.1015	0.1016
Lapatinib	Points from 8 dose regions	0.4552	0.4688	0.5052	0.0579	0.0573	0.048
	Distributions from 8 dose regions (KL divergence)		0.4524	0.5056		0.0578	0.0481
	Distributions from 8 dose regions (Hellinger Distance)		0.4633	**0.5285**		0.0578	**0.0474**
Nutlin-3	Points from 8 dose regions	0.0542	**0.124**	0.0648	0.0356	0.0354	**0.0302**
	Distributions from 8 dose regions (KL divergence)		0.0993	0.0519		0.0353	0.0302
	Distributions from 8 dose regions (Hellinger Distance)		0.0796	0.0238		0.0354	0.0304
Paclitaxel	Points from 8 dose regions	0.3934	0.3905	0.4251	0.1215	0.1216	0.1241
	Distributions from 8 dose regions (KL divergence)		0.3773	0.4022		0.1207	0.1236
	Distributions from 8 dose regions (Hellinger Distance)		0.3932	0.4266		0.1209	0.1238
PD-0332991	Points from 8 dose regions	0.4115	0.4394	0.4180	0.0512	0.0503	**0.0459**
	Distributions from 8 dose regions (KL divergence)		0.4226	0.3968		0.0506	0.0463
	Distributions from 8 dose regions (Hellinger Distance)		**0.4445**	0.4127		0.0503	0.0463
PF2341066 /Crizotinib	Points from 8 dose regions	0.2931	**0.3266**	0.3095	0.0459	0.0452	**0.0372**
	Distributions from 8 dose regions (KL divergence)		0.2742	0.2434		0.0462	0.0378
	Distributions from 8 dose regions (Hellinger Distance)		0.2281	0.1755		0.0464	0.0384
PHA-665752	Points from 8 dose regions	0.1695	0.1902	0.1588	0.0469	0.0464	**0.0375**
	Distributions from 8 dose regions (KL divergence)		**0.2031**	0.1898		0.0457	0.0375
	Distributions from 8 dose regions (Hellinger Distance)		0.198	0.1592		0.0457	0.0378
Sorafenib	Points from 8 dose regions	0.3755	**0.3824**	0.3668	0.0384	0.0383	**0.0321**
	Distributions from 8 dose regions (KL divergence)		0.3390	0.3023		0.0391	0.0328
	Distributions from 8 dose regions (Hellinger Distance)		0.3392	0.3143		0.0392	0.0327
AEW541	Points from 8 dose regions	0.2757	0.2619	**0.3073**	0.0545	0.0548	**0.0487**
	Distributions from 8 dose regions (KL divergence)		0.2331	0.2758		0.0554	0.0493

Table S1: Correlation coefficient and Mean Absolute Error (MAE) between actual and predicted drug sensitivities (AUC) for 3 methods; RF, FRFL and FRF with three different model constructions. In FRFL and FRF, node cost is calculated using 8 dose regions. Here, results of all 24 drugs of CCLE database are shown.

Drug Name	FRF node cost criteria	Correlation Coefficient			Mean Absolute Error		
		RF	FRFL	FRF	RF	FRFL	FRF
	Distributions from 8 dose regions (Hellinger Distance)		0.2409	0.2875		0.0553	0.0491
Irinotecan	Points from 8 dose regions	0.519	0.5234	**0.5344**	0.0881	0.0875	0.1021
	Distributions from 8 dose regions (KL divergence)		0.528	0.5321		**0.0872**	0.1021
	Distributions from 8 dose regions (Hellinger Distance)		0.5285	0.5332		0.0883	0.1029
L-685458	Points from 8 dose regions	0.3611	0.3754	**0.4000**	0.0416	0.0412	**0.0295**
	Distributions from 8 dose regions (KL divergence)		0.3571	0.3810		0.0416	0.0302
	Distributions from 8 dose regions (Hellinger Distance)		0.3553	0.3758		0.0417	0.0301
LBW242	Points from 8 dose regions	0.1140	0.1217	**0.1377**	0.0563	0.0556	**0.0517**
	Distributions from 8 dose regions (KL divergence)		0.0662	0.1032		0.0558	0.0526
	Distributions from 8 dose regions (Hellinger Distance)		0.0661	0.1198		0.0562	0.0528
Panobinostat	Points from 8 dose regions	0.5239	0.5335	0.5584	**0.0640**	0.0641	0.0724
	Distributions from 8 dose regions (KL divergence)		0.5101	0.5374		0.0651	0.0739
	Distributions from 8 dose regions (Hellinger Distance)		0.5388	**0.5611**		0.0643	0.0731
RAF265	Points from 8 dose regions	0.3739	0.3992	**0.4149**	0.0687	**0.0684**	0.0716
	Distributions from 8 dose regions (KL divergence)		0.3557	0.3735		0.0690	0.0724
	Distributions from 8 dose regions (Hellinger Distance)		0.3005	0.3123		0.0704	0.0738
TKI258	Points from 8 dose regions	0.3799	**0.3986**	0.3831	0.0512	0.0506	**0.0447**
	Distributions from 8 dose regions (KL divergence)		0.3408	0.3041		0.0521	0.0461
	Distributions from 8 dose regions (Hellinger Distance)		0.3377	0.3261		0.0523	0.0457
Topotecan	Points from 8 dose regions	0.5122	0.5006	0.5297	0.1055	0.1061	0.1161
	Distributions from 8 dose regions (KL divergence)		0.5138	**0.5342**		**0.1053**	0.1158
	Distributions from 8 dose regions (Hellinger Distance)		0.4983	0.5255		0.1069	0.1176
ZD-6474	Points from 8 dose regions	0.2428	0.2696	0.2735	0.0641	0.0634	**0.0581**
	Distributions from 8 dose regions (KL divergence)		0.2602	**0.2842**		0.0634	0.0582
	Distributions from 8 dose regions (Hellinger Distance)		0.185	0.2107		0.0643	0.059

Table S2: Mean Absolute Error (MAE) between actual and predicted drug sensitivities (AUC and different IC's) for 2 methods; RF and FRF. In FRF, node cost is calculated using 8 IC regions. Here, results of all 140 drugs of GDSC database $v5$ are shown.

Drug Name	Model	AUC	IC_{10}	IC_{20}	IC_{30}	IC_{40}	IC_{50}	IC_{60}	IC_{70}	IC_{80}	Average
Erlotinib	RF	0.0596	2.0831	1.7472	1.5039	1.3291	1.1948	1.0692	1.0133	1.0304	1.3714
	FRF	0.0486	1.9813	1.6597	1.4382	1.2694	1.1357	1.0361	0.9867	1.0095	1.3146
Rapamycin	RF	0.064	4.3771	3.4771	2.937	2.5294	2.2	2.0355	2.0207	2.5359	2.7641

Table S2: Mean Absolute Error (MAE) between actual and predicted drug sensitivities (AUC and different IC's) for 2 methods; RF and FRF. In FRF, node cost is calculated using 8 IC regions. Here, results of all 140 drugs of GDSC database $v5$ are shown.

Drug Name	Model	AUC	IC_{10}	IC_{20}	IC_{30}	IC_{40}	IC_{50}	IC_{60}	IC_{70}	IC_{80}	Average
	FRF	0.0636	4.3905	3.4525	2.8895	2.4642	2.1379	1.9446	2.0046	2.4707	2.7193
Sunitinib	RF	0.0963	1.5494	1.5297	1.5542	1.6105	1.6518	1.7013	1.775	1.8728	1.6556
	FRF	0.0902	1.5306	1.5119	1.5378	1.575	1.6276	1.6812	1.7428	1.8372	1.6305
PHA-665752	RF	0.037	1.4403	1.2665	1.1492	1.0658	1.0002	0.9555	0.9539	0.9485	1.0975
	FRF	0.0259	1.3522	1.2051	1.0999	1.0149	0.9546	0.9054	0.8954	0.9097	1.0422
MG-132	RF	0.1246	1.6207	1.6688	1.7445	1.783	1.8549	1.9289	2.0313	2.1509	1.8479
	FRF	0.107	1.6062	1.6479	1.6968	1.7541	1.8117	1.8794	1.9619	2.0857	1.8055
Paclitaxel	RF	0.1427	1.8698	1.8716	1.8982	1.947	1.9976	2.0908	2.1783	2.3253	2.0223
	FRF	0.1435	1.7694	1.763	1.8013	1.8616	1.9243	2.0036	2.1211	2.2762	1.9401
Cyclopamine	RF	0.0315	1.3288	1.2346	1.188	1.1736	1.155	1.1749	1.2055	1.2627	1.2154
	FRF	0.0274	1.34	1.2443	1.1874	1.1624	1.1541	1.1556	1.1646	1.2096	1.2022
AZ628	RF	0.133	2.5134	2.1669	1.9748	1.8824	1.823	1.8044	1.8521	1.9362	1.9942
	FRF	0.1203	2.446	2.1155	1.9411	1.8337	1.7714	1.7544	1.7966	1.8978	1.9446
Sorafenib	RF	0.0766	1.5314	1.4777	1.4452	1.4407	1.4627	1.4575	1.4845	1.532	1.4789
	FRF	0.0662	1.5193	1.4681	1.4438	1.4394	1.4388	1.4417	1.4622	1.5037	1.4646
VX-680	RF	0.128	2.5637	2.3388	2.2608	2.1607	2.1121	2.0611	2.0323	2.0098	2.1924
	FRF	0.12	2.526	2.3258	2.2177	2.1569	2.0957	2.0445	2.0051	1.9705	2.1678
Imatinib	RF	0.0383	1.2762	1.1374	1.07	0.9765	0.9454	0.9043	0.872	0.8502	1.004
	FRF	0.0258	1.1553	1.0502	0.9733	0.9056	0.8562	0.8255	0.8	0.7784	0.9181
NVP-TAE684	RF	0.1156	1.7224	1.6634	1.6829	1.7587	1.8194	1.8944	2.0052	2.1901	1.8421
	FRF	0.111	1.7162	1.656	1.673	1.7253	1.795	1.8872	2.0037	2.1722	1.8286
PF-02341066	RF	0.0475	1.2559	1.1949	1.1595	1.1705	1.1532	1.1884	1.1808	1.1968	1.1875
	FRF	0.0364	1.2281	1.1682	1.1354	1.1268	1.1234	1.125	1.1286	1.1339	1.1462
AZD-0530	RF	0.0661	1.7654	1.6408	1.5783	1.5453	1.5295	1.5384	1.5438	1.5804	1.5902
	FRF	0.0543	1.7413	1.6184	1.5425	1.494	1.4786	1.4608	1.4598	1.4899	1.5357
S-Trityl-L-cysteine	RF	0.0844	1.3216	1.3881	1.4569	1.5297	1.6323	1.7073	1.8311	1.9878	1.6069
	FRF	0.0857	1.2496	1.2909	1.3527	1.4335	1.5304	1.6421	1.7723	1.9423	1.5267
Z-LLNle-CHO	RF	0.0821	1.1943	1.2622	1.3096	1.3575	1.419	1.506	1.5874	1.7042	1.4175
	FRF	0.0776	1.2078	1.2557	1.3103	1.3656	1.4311	1.498	1.5824	1.7033	1.4193
Dasatinib	RF	0.1706	3.5646	3.0769	2.8374	2.7091	2.6161	2.5967	2.6919	2.8865	2.8724
	FRF	0.1533	3.4158	2.8774	2.5761	2.4423	2.3922	2.4175	2.5577	2.8209	2.6875
GNF-2	RF	0.0288	1.1946	1.0841	1.0022	0.9293	0.8772	0.805	0.7644	0.726	0.9229
	FRF	0.0194	1.1075	1.0125	0.9392	0.8756	0.815	0.7561	0.7052	0.6711	0.8603
CGP-60474	RF	0.1135	1.0519	1.1472	1.2349	1.3366	1.4259	1.5384	1.637	1.8148	1.3983
	FRF	0.11	1.037	1.1013	1.176	1.2478	1.3304	1.4262	1.5422	1.6967	1.3197
CGP-082996	RF	0.0499	1.3682	1.2746	1.2494	1.2615	1.2897	1.3178	1.3822	1.4746	1.3273
	FRF	0.0483	1.3642	1.2538	1.2258	1.2308	1.2492	1.2857	1.3428	1.4244	1.2971
A-770041	RF	0.1158	2.3238	2.0588	1.9727	1.9075	1.9085	1.9381	1.9732	2.0792	2.0202
	FRF	0.1057	2.3063	2.0748	1.961	1.8997	1.8858	1.9116	1.9702	2.0925	2.0127
WH-4-023	RF	0.1482	3.4064	2.961	2.6643	2.5482	2.3909	2.325	2.2569	2.2913	2.6055
	FRF	0.1328	3.2713	2.8204	2.5265	2.3121	2.2125	2.153	2.1514	2.2259	2.4591
WZ-1-84	RF	0.0514	1.4484	1.3295	1.2695	1.2642	1.264	1.2992	1.3357	1.3759	1.3233
	FRF	0.0459	1.4604	1.3367	1.2716	1.2381	1.2306	1.2573	1.3035	1.3701	1.3085
BI-2536	RF	0.1117	1.4583	1.5436	1.5907	1.6808	1.7689	1.8626	1.9685	2.1153	1.7486
	FRF	0.1116	1.3783	1.4435	1.5221	1.6042	1.6938	1.7954	1.9141	2.0728	1.678
BMS-536924	RF	0.1267	2.0026	1.8233	1.7752	1.8229	1.8784	1.9658	2.0645	2.2417	1.9468
	FRF	0.1229	1.9668	1.805	1.7773	1.8113	1.8687	1.9507	2.0696	2.2405	1.9362
BMS-509744	RF	0.061	1.1916	1.1434	1.1819	1.2403	1.3262	1.4328	1.5584	1.7181	1.3491
	FRF	0.0598	1.1735	1.1189	1.1543	1.2289	1.3097	1.4059	1.5275	1.6834	1.3253
CMK	RF	0.0542	1.2626	1.2502	1.2695	1.2988	1.3715	1.4291	1.5457	1.6627	1.3863
	FRF	0.052	1.2608	1.2323	1.2579	1.2972	1.3541	1.4314	1.53	1.6566	1.3775
Pyrimethamine	RF	0.1023	1.8621	1.7736	1.7716	1.7731	1.8369	1.8816	1.9761	2.1094	1.8731
	FRF	0.0977	1.8691	1.7703	1.7436	1.7442	1.7803	1.8403	1.927	2.0521	1.8409

Table S2: Mean Absolute Error (MAE) between actual and predicted drug sensitivities (AUC and different IC's) for 2 methods; RF and FRF. In FRF, node cost is calculated using 8 IC regions. Here, results of all 140 drugs of GDSC database $v5$ are shown.

Drug Name	Model	AUC	IC_{10}	IC_{20}	IC_{30}	IC_{40}	IC_{50}	IC_{60}	IC_{70}	IC_{80}	Average
JW-7-52-1	RF	0.1656	1.7563	1.7574	1.8373	1.9039	2.0276	2.1625	2.331	2.6052	2.0476
	FRF	0.1624	1.7331	1.6977	1.7539	1.828	1.9207	2.0476	2.2316	2.5101	1.9654
A-443654	RF	0.1041	1.1877	1.2548	1.3428	1.4252	1.525	1.6264	1.7499	1.9333	1.5056
	FRF	0.1098	1.1481	1.2038	1.2673	1.3345	1.4148	1.5074	1.6219	1.7743	1.409
GW843682X	RF	0.1313	1.4825	1.5624	1.6745	1.8111	1.9213	2.0503	2.2123	2.4399	1.8943
	FRF	0.1282	1.3539	1.4181	1.5279	1.6495	1.7819	1.9227	2.0874	2.305	1.7558
MS-275	RF	0.1071	1.4331	1.3756	1.4381	1.5353	1.6439	1.8035	1.9861	2.2498	1.6832
	FRF	0.1101	1.4131	1.3375	1.3661	1.4284	1.5403	1.687	1.8716	2.1332	1.5971
Parthenolide	RF	0.0292	1.3288	1.3005	1.2851	1.2953	1.3239	1.3206	1.3652	1.4048	1.328
	FRF	0.0252	1.3231	1.2926	1.2821	1.2779	1.2901	1.3136	1.346	1.389	1.3143
KIN001-135	RF	0.0109	0.9776	0.8199	0.7239	0.6385	0.5848	0.5464	0.5196	0.516	0.6658
	FRF	0.0084	0.9109	0.7773	0.6848	0.6167	0.5716	0.5382	0.5178	0.5215	0.6424
TGX221	RF	0.0423	2.1298	1.7658	1.5722	1.409	1.2886	1.206	1.1504	1.1496	1.4589
	FRF	0.0373	2.0421	1.7232	1.499	1.3511	1.2383	1.1534	1.1117	1.1188	1.4047
Bortezomib	RF	0.1612	1.1812	1.2946	1.4282	1.551	1.6545	1.7758	1.9099	2.0991	1.6118
	FRF	0.1573	1.1741	1.2949	1.4047	1.5082	1.6134	1.7261	1.8537	2.0156	1.5738
XMD8-85	RF	0.061	1.2746	1.2539	1.2722	1.3208	1.3816	1.4359	1.5091	1.6198	1.3835
	FRF	0.0612	1.2595	1.2201	1.2349	1.2819	1.3317	1.3892	1.4667	1.5738	1.3447
Roscovitine	RF	0.0323	1.2444	1.2559	1.2824	1.3096	1.3652	1.4049	1.4788	1.5651	1.3633
	FRF	0.0359	1.2161	1.2168	1.2533	1.2978	1.3431	1.3985	1.4652	1.5597	1.3438
Salubrinal	RF	0.0412	1.1609	1.1683	1.2238	1.2452	1.3132	1.3688	1.4493	1.5688	1.3123
	FRF	0.0453	1.1214	1.0955	1.141	1.1998	1.2623	1.3363	1.4309	1.5557	1.2679
Lapatinib	RF	0.035	1.6051	1.438	1.3449	1.2572	1.1899	1.1476	1.1089	1.098	1.2737
	FRF	0.0286	1.5788	1.4157	1.3059	1.2172	1.143	1.089	1.0585	1.0568	1.2331
GSK269962A	RF	0.0607	2.4652	2.0184	1.8144	1.6904	1.65	1.6292	1.7006	1.8324	1.8501
	FRF	0.0605	2.4226	1.9997	1.7799	1.6436	1.6047	1.6241	1.6956	1.8423	1.8266
Doxorubicin	RF	0.146	1.5127	1.464	1.431	1.4189	1.4387	1.4453	1.4883	1.5702	1.4712
	FRF	0.1482	1.5085	1.4499	1.4216	1.4113	1.4209	1.4414	1.4862	1.5749	1.4643
Etoposide	RF	0.145	1.8283	1.7765	1.7773	1.7961	1.8403	1.8841	1.9478	2.0716	1.8653
	FRF	0.1431	1.8283	1.7641	1.7639	1.782	1.8172	1.8627	1.9266	2.0524	1.8496
Gemcitabine	RF	0.1459	3.9367	3.4848	3.2466	3.0895	3.0124	2.9827	2.9557	3.0874	3.2245
	FRF	0.2146	3.896	3.4867	3.2414	3.0957	3.0027	2.9604	2.9728	3.0809	3.2171
Mitomycin C	RF	0.1171	1.9372	1.7892	1.7216	1.6468	1.5763	1.5434	1.4947	1.4565	1.6457
	FRF	0.166	1.9319	1.7791	1.6884	1.6264	1.5792	1.5401	1.5012	1.4665	1.6391
Vinorelbine	RF	0.1313	1.3498	1.3781	1.4062	1.4723	1.5399	1.6081	1.7286	1.8578	1.5426
	FRF	0.1311	1.339	1.3442	1.3768	1.4235	1.4918	1.575	1.6774	1.8263	1.5067
NSC-87877	RF	0.0182	0.9975	0.9346	0.9005	0.8663	0.8418	0.8251	0.8191	0.8005	0.8732
	FRF	0.0143	0.9655	0.8984	0.8544	0.8233	0.7978	0.7762	0.7607	0.7475	0.828
Bicalutamide	RF	0.0043	0.5256	0.4937	0.4716	0.448	0.4318	0.4067	0.3866	0.3649	0.4411
	FRF	0.0034	0.5043	0.4732	0.4495	0.429	0.4099	0.3916	0.372	0.3497	0.4224
QS11	RF	0.0659	1.5704	1.5348	1.5367	1.5418	1.5611	1.5827	1.6017	1.6525	1.5727
	FRF	0.0657	1.5393	1.5121	1.5097	1.5186	1.5372	1.562	1.5936	1.641	1.5517
Midostaurin	RF	0.059	1.3132	1.3397	1.3573	1.3883	1.4276	1.4713	1.5155	1.594	1.4259
	FRF	0.0579	1.2857	1.3034	1.3379	1.3728	1.4107	1.4553	1.5075	1.5754	1.4061
CHIR-99021	RF	0.0275	1.3387	1.2564	1.1942	1.1607	1.1124	1.0686	1.0328	0.9916	1.1444
	FRF	0.0214	1.2826	1.2084	1.1523	1.104	1.0614	1.0224	0.9842	0.9374	1.0941
AP-24534	RF	0.0598	1.8811	1.7809	1.7523	1.7353	1.734	1.7353	1.7346	1.7877	1.7677
	FRF	0.0533	1.8183	1.7588	1.7281	1.7159	1.7161	1.7172	1.7198	1.7526	1.7409
AZD6482	RF	0.0638	1.9669	1.706	1.5726	1.4941	1.4239	1.3791	1.3598	1.3879	1.5363
	FRF	0.062	1.9497	1.7093	1.5707	1.4812	1.419	1.3785	1.3618	1.3913	1.5327
JNK-9L	RF	0.1081	0.9043	0.8835	0.8868	0.9125	0.9576	1.0019	1.0787	1.1741	0.9749
	FRF	0.1117	0.9105	0.8647	0.8666	0.8872	0.9211	0.965	1.0291	1.1202	0.9456
PF-562271	RF	0.0412	1.2589	1.209	1.1953	1.1869	1.2109	1.2469	1.2855	1.3578	1.2439

Table S2: Mean Absolute Error (MAE) between actual and predicted drug sensitivities (AUC and different IC's) for 2 methods; RF and FRF. In FRF, node cost is calculated using 8 IC regions. Here, results of all 140 drugs of GDSC database $v5$ are shown.

Drug Name	Model	AUC	IC_{10}	IC_{20}	IC_{30}	IC_{40}	IC_{50}	IC_{60}	IC_{70}	IC_{80}	Average
	FRF	0.0424	1.2454	1.1901	1.1721	1.1719	1.1927	1.2285	1.2812	1.356	1.2297
DMOG	RF	0.1312	1.282	1.2228	1.2297	1.2427	1.2814	1.3371	1.4255	1.5572	1.3223
	FRF	0.133	1.3027	1.2391	1.2319	1.2473	1.2736	1.323	1.4053	1.5426	1.3207
FTI-277	RF	0.017	0.9804	0.9174	0.8753	0.838	0.8089	0.7695	0.7407	0.721	0.8314
	FRF	0.0145	0.9717	0.9137	0.8681	0.8288	0.7917	0.7599	0.7315	0.701	0.8208
OSU-03012	RF	0.1211	1.5365	1.5877	1.6314	1.6724	1.7218	1.774	1.8417	1.9437	1.7136
	FRF	0.1179	1.4924	1.5354	1.5826	1.6361	1.6905	1.7535	1.8317	1.9336	1.682
Shikonin	RF	0.1557	1.2026	1.2308	1.2686	1.312	1.3616	1.3992	1.4636	1.5541	1.3491
	FRF	0.1529	1.1958	1.2223	1.247	1.2749	1.3038	1.3369	1.3785	1.4357	1.2994
AKT inhibitor VIII	RF	0.0424	1.44	1.345	1.2913	1.2431	1.2194	1.1865	1.1679	1.1553	1.2561
	FRF	0.0417	1.453	1.3522	1.2966	1.2561	1.2208	1.1905	1.1667	1.1553	1.2614
Embelin	RF	0.0798	0.8046	0.8379	0.8703	0.9357	0.9864	1.0594	1.1543	1.2867	0.9919
	FRF	0.0834	0.7968	0.8114	0.8484	0.8979	0.9553	1.0227	1.1102	1.2364	0.9599
FH535	RF	0.0993	1.0428	0.951	0.9502	0.9915	1.0625	1.1634	1.3185	1.519	1.1249
	FRF	0.1035	1.041	0.9551	0.9405	0.976	1.043	1.1383	1.2634	1.4528	1.1012
PAC-1	RF	0.0419	1.279	1.2928	1.3061	1.3224	1.3504	1.3731	1.4113	1.4787	1.3517
	FRF	0.0374	1.2726	1.2758	1.2896	1.3037	1.3209	1.3453	1.3737	1.4199	1.3252
IPA-3	RF	0.0718	1.4249	1.493	1.5406	1.5882	1.633	1.6929	1.7499	1.8335	1.6195
	FRF	0.062	1.4351	1.4978	1.5429	1.5839	1.6248	1.6678	1.7185	1.7867	1.6072
GSK-650394	RF	0.083	1.9389	1.8127	1.7379	1.6825	1.6365	1.6407	1.6421	1.6717	1.7204
	FRF	0.0821	1.9194	1.7913	1.7168	1.6742	1.6502	1.643	1.652	1.6793	1.7158
BAY 61-3606	RF	0.0847	1.4156	1.3745	1.3676	1.3639	1.3851	1.4141	1.4754	1.5352	1.4164
	FRF	0.084	1.3702	1.3379	1.3305	1.3458	1.3714	1.4056	1.4533	1.5239	1.3923
Thapsigargin	RF	0.1443	2.4606	2.2228	2.125	2.1017	2.1009	2.1735	2.3333	2.5558	2.2592
	FRF	0.2006	2.4415	2.2248	2.1392	2.099	2.1106	2.167	2.2942	2.5073	2.2479
Obatoclax Mesylate	RF	0.1403	2.1192	1.8568	1.726	1.6432	1.6261	1.6296	1.6731	1.7857	1.7575
	FRF	0.1898	2.0813	1.8454	1.7201	1.6484	1.6151	1.6267	1.6781	1.7878	1.7503
BMS-754807	RF	0.095	2.2446	1.9272	1.7603	1.6571	1.6255	1.6202	1.6669	1.7564	1.7823
	FRF	0.0949	2.1717	1.9078	1.7596	1.6652	1.613	1.6063	1.6544	1.7682	1.7683
OSI-906	RF	0.0694	2.1853	1.9067	1.7479	1.6237	1.5188	1.4414	1.341	1.3061	1.6339
	FRF	0.0644	2.166	1.9151	1.7471	1.6167	1.5058	1.4085	1.3314	1.2881	1.6223
Bexarotene	RF	0.0379	1.6856	1.4569	1.3395	1.2513	1.1976	1.2045	1.2582	1.3605	1.3443
	FRF	0.0384	1.6478	1.4519	1.337	1.2485	1.2035	1.2111	1.2655	1.3683	1.3417
Bleomycin	RF	0.1465	2.8279	2.6156	2.485	2.4248	2.3984	2.4	2.4083	2.5034	2.5079
	FRF	0.1869	2.7953	2.5831	2.4724	2.4019	2.371	2.3788	2.4123	2.5022	2.4896
LFM-A13	RF	0.0202	1.1245	0.9644	0.8699	0.804	0.758	0.7283	0.7126	0.7228	0.8356
	FRF	0.0184	1.1058	0.9607	0.8679	0.8057	0.7607	0.7251	0.7052	0.7068	0.8298
AUY922	RF	0.1312	1.1848	1.2117	1.2561	1.2907	1.3405	1.3907	1.4608	1.5617	1.3371
	FRF	0.1297	1.1737	1.1872	1.2198	1.2552	1.2975	1.3482	1.4148	1.5174	1.3017
Bryostatin 1	RF	0.0241	1.2331	1.1275	1.0628	0.9986	0.9394	0.888	0.8339	0.7944	0.9847
	FRF	0.0204	1.215	1.1204	1.0495	0.9872	0.9293	0.873	0.8234	0.7719	0.9712
Pazopanib	RF	0.0561	1.5966	1.4852	1.4074	1.3737	1.3422	1.3316	1.3162	1.3093	1.3953
	FRF	0.0534	1.5933	1.4671	1.4006	1.3512	1.3164	1.2917	1.2792	1.2772	1.3721
LAQ824	RF	0.1171	1.0134	0.9509	0.9358	0.9405	0.9547	0.984	1.0351	1.1238	0.9923
	FRF	0.119	1.0096	0.9521	0.9311	0.9295	0.9428	0.9638	1.0025	1.0748	0.9758
Epothilone B	RF	0.1433	1.6792	1.636	1.6373	1.659	1.7077	1.737	1.812	1.9295	1.7247
	FRF	0.1451	1.676	1.6324	1.6309	1.6429	1.6752	1.7219	1.7882	1.904	1.7089
GSK-1904529A	RF	0.014	0.9711	0.9024	0.855	0.8122	0.7676	0.7378	0.6971	0.6607	0.8005
	FRF	0.0116	0.9495	0.8884	0.8393	0.7958	0.7546	0.7192	0.6828	0.6416	0.7839
Tipifarnib	RF	0.1036	2.2791	1.8959	1.7326	1.6667	1.657	1.7012	1.8276	2.0671	1.8534
	FRF	0.1211	2.2918	1.9192	1.7418	1.6617	1.6534	1.698	1.8189	2.0461	1.8539
AS601245	RF	0.0666	1.3894	1.3622	1.383	1.3969	1.4299	1.4694	1.5341	1.6179	1.4478
	FRF	0.0666	1.36	1.3358	1.3415	1.363	1.3979	1.4462	1.5136	1.6098	1.421

Table S2: Mean Absolute Error (MAE) between actual and predicted drug sensitivities (AUC and different IC's) for 2 methods; RF and FRF. In FRF, node cost is calculated using 8 IC regions. Here, results of all 140 drugs of GDSC database $v5$ are shown.

Drug Name	Model	AUC	IC_{10}	IC_{20}	IC_{30}	IC_{40}	IC_{50}	IC_{60}	IC_{70}	IC_{80}	Average
AICAR	RF	0.0639	1.0269	1.0315	1.0612	1.0972	1.1428	1.1881	1.2393	1.3417	1.1411
	FRF	0.0668	1.032	1.0296	1.0486	1.077	1.1155	1.1654	1.2304	1.3265	1.1281
Camptothecin	RF	0.1269	1.5973	1.5151	1.5145	1.5219	1.5673	1.6265	1.6957	1.8158	1.6067
	FRF	NaN	1.6203	1.5256	1.5084	1.5273	1.5692	1.6261	1.7031	1.8225	1.6128
Vinblastine	RF	0.1117	1.3215	1.2419	1.2199	1.2175	1.2354	1.2892	1.3667	1.4953	1.2984
	FRF	0.12	1.3417	1.2558	1.2267	1.2257	1.2415	1.2834	1.3566	1.4724	1.3005
Cisplatin	RF	0.0592	1.1523	1.1402	1.1525	1.1805	1.2153	1.255	1.3148	1.3796	1.2238
	FRF	0.0607	1.1455	1.1276	1.1393	1.1709	1.2068	1.2511	1.3039	1.3755	1.2151
Cytarabine	RF	0.0991	1.7843	1.6124	1.5181	1.4816	1.4744	1.5027	1.5626	1.6881	1.578
	FRF	0.1005	1.7876	1.61	1.5156	1.4714	1.4611	1.4841	1.5435	1.6621	1.5669
Docetaxel	RF	0.0952	1.4072	1.333	1.295	1.2839	1.2871	1.3209	1.3588	1.4814	1.3459
	FRF	0.0963	1.4254	1.3339	1.2861	1.2713	1.2738	1.3038	1.3626	1.4675	1.3406
Methotrexate	RF	0.0677	1.6933	1.6202	1.5865	1.5476	1.5207	1.4989	1.4832	1.4829	1.5542
	FRF	0.0582	1.6171	1.5491	1.5046	1.4724	1.4463	1.4331	1.4225	1.418	1.4829
ATRA	RF	0.0403	2.1161	1.7504	1.5301	1.3344	1.1747	1.0602	1.0062	1.0221	1.3743
	FRF	0.0372	1.9996	1.6972	1.49	1.3105	1.1537	1.0337	0.9826	1.0161	1.3354
Gefitinib	RF	0.0314	1.3019	1.1917	1.1168	1.0529	0.9942	0.9415	0.8987	0.8856	1.0479
	FRF	0.0262	1.2569	1.1625	1.0939	1.0359	0.9824	0.9296	0.8841	0.8653	1.0263
ABT-263	RF	0.087	1.752	1.6846	1.6421	1.6277	1.6268	1.6465	1.6753	1.7377	1.6741
	FRF	0.081	1.7347	1.6664	1.6356	1.6158	1.6015	1.6059	1.6261	1.6819	1.646
Vorinostat	RF	0.0868	0.7416	0.7226	0.7217	0.7418	0.7701	0.8043	0.8558	0.9407	0.7873
	FRF	0.0938	0.7441	0.7183	0.7217	0.7374	0.7612	0.7935	0.8382	0.9116	0.7782
Nilotinib	RF	0.0272	1.2416	1.2039	1.176	1.1554	1.1211	1.0933	1.0651	1.0384	1.1369
	FRF	0.0214	1.1982	1.1573	1.1257	1.0969	1.0686	1.0399	1.0093	0.9726	1.0835
RDEA119	RF	0.0915	2.0818	1.8439	1.7375	1.6584	1.6133	1.5826	1.5932	1.6336	1.718
	FRF	0.0872	2.0382	1.8271	1.6984	1.6085	1.5616	1.5375	1.5337	1.5507	1.6694
CI-1040	RF	0.0801	1.7216	1.5116	1.3926	1.3309	1.2916	1.2795	1.3112	1.3987	1.4047
	FRF	0.0791	1.6772	1.4861	1.3904	1.3296	1.2948	1.2845	1.313	1.3826	1.3948
Temsirolimus	RF	0.0605	3.107	2.4579	2.0394	1.7022	1.4974	1.4061	1.4458	1.6783	1.9168
	FRF	0.1137	3.0387	2.3981	1.9973	1.7073	1.5012	1.4104	1.4452	1.6829	1.8976
AZD-2281	RF	0.028	1.1966	1.1539	1.1184	1.0839	1.07	1.0567	1.036	1.0143	1.0912
	FRF	0.023	1.1603	1.1175	1.0858	1.0572	1.0343	1.0155	0.9952	0.9727	1.0548
ABT-888	RF	0.0144	0.9447	0.8848	0.8447	0.7993	0.7594	0.719	0.685	0.6296	0.7833
	FRF	0.0114	0.8949	0.8407	0.7989	0.7613	0.7255	0.6888	0.6488	0.6016	0.7451
Bosutinib	RF	0.0608	1.588	1.5523	1.5198	1.506	1.4876	1.4802	1.4847	1.4832	1.5127
	FRF	0.0567	1.5754	1.5287	1.5083	1.4951	1.4841	1.4743	1.4678	1.4686	1.5003
Lenalidomide	RF	0.016	1.0052	0.9387	0.8897	0.8407	0.8038	0.7576	0.7035	0.6485	0.8235
	FRF	0.0129	0.9652	0.9017	0.8523	0.8085	0.7671	0.7248	0.6783	0.6235	0.7902
Axitinib	RF	0.0435	1.3575	1.3153	1.2879	1.2808	1.2663	1.2524	1.2651	1.2768	1.2878
	FRF	0.0383	1.3407	1.3041	1.2828	1.2651	1.251	1.2418	1.2396	1.2482	1.2717
AZD7762	RF	0.1047	1.2514	1.1954	1.1751	1.1599	1.1632	1.1743	1.2039	1.2718	1.1994
	FRF	0.1076	1.2601	1.2016	1.1752	1.1639	1.158	1.1645	1.1946	1.2555	1.1967
GW 441756	RF	0.0316	1.599	1.3779	1.2191	1.1016	0.9982	0.9177	0.8564	0.8428	1.1141
	FRF	0.026	1.5017	1.3113	1.1758	1.067	0.9759	0.898	0.8428	0.8363	1.0761
CEP-701	RF	0.0924	1.6556	1.4444	1.342	1.2731	1.2387	1.2494	1.303	1.4221	1.366
	FRF	0.1027	1.6428	1.4423	1.3325	1.2636	1.2273	1.2334	1.2889	1.412	1.3553
SB 216763	RF	0.0198	1.0793	1.0495	1.0317	0.9973	0.9823	0.9663	0.9409	0.9195	0.9958
	FRF	0.0164	1.0359	1.0057	0.9814	0.9585	0.9361	0.9129	0.888	0.8593	0.9472
17-AAG	RF	0.1087	1.678	1.6017	1.5755	1.547	1.5519	1.5611	1.6035	1.671	1.5987
	FRF	0.1058	1.662	1.5823	1.5418	1.5178	1.5106	1.5166	1.5492	1.6233	1.5629
VX-702	RF	0.0182	1.0286	0.94	0.8731	0.8194	0.7664	0.7161	0.6633	0.5946	0.8002
	FRF	0.0144	0.9724	0.8959	0.8398	0.7904	0.7437	0.6956	0.6431	0.5797	0.7701
AMG-706	RF	0.0248	1.2973	1.1542	1.0645	0.9935	0.9026	0.8418	0.7904	0.7485	0.9741

Table S2: Mean Absolute Error (MAE) between actual and predicted drug sensitivities (AUC and different IC's) for 2 methods; RF and FRF. In FRF, node cost is calculated using 8 IC regions. Here, results of all 140 drugs of GDSC database $v5$ are shown.

Drug Name	Model	AUC	IC_{10}	IC_{20}	IC_{30}	IC_{40}	IC_{50}	IC_{60}	IC_{70}	IC_{80}	Average
	FRF	0.0196	1.2103	1.0937	1.0077	0.9318	0.862	0.8004	0.7474	0.7164	0.9212
KU-55933	RF	0.028	1.1351	1.1014	1.0778	1.0601	1.054	1.0355	1.0323	1.0445	1.0676
	FRF	0.0256	1.1129	1.0832	1.0653	1.0508	1.0366	1.0265	1.0212	1.0236	1.0525
Elesclomol	RF	0.141	1.4571	1.4762	1.4966	1.5546	1.6021	1.657	1.7432	1.8698	1.6071
	FRF	0.1409	1.4824	1.4842	1.5052	1.5333	1.5754	1.6297	1.7157	1.8372	1.5954
BIBW2992	RF	0.0455	1.68	1.4938	1.3805	1.2748	1.1871	1.0968	1.0267	0.9668	1.2633
	FRF	0.0365	1.5605	1.3988	1.2878	1.1936	1.1062	1.0248	0.9605	0.9066	1.1798
GDC-0449	RF	0.0189	1.0277	1.005	0.9847	0.9597	0.9458	0.9244	0.8995	0.8784	0.9531
	FRF	0.016	1.0153	0.9884	0.9657	0.9442	0.9233	0.9023	0.8801	0.8541	0.9342
PLX4720	RF	0.0483	1.5692	1.4689	1.4215	1.3678	1.3222	1.2913	1.2586	1.2538	1.3692
	FRF	0.0414	1.5306	1.4469	1.3894	1.3402	1.2967	1.2556	1.2247	1.212	1.337
BX-795	RF	0.062	1.0441	1.0744	1.0978	1.1351	1.1623	1.2135	1.2705	1.3425	1.1675
	FRF	0.0674	1.0347	1.0469	1.0758	1.1086	1.1525	1.2035	1.2639	1.3461	1.154
NU-7441	RF	0.0275	1.1714	1.1346	1.1131	1.0902	1.0786	1.0547	1.0453	1.0503	1.0923
	FRF	0.0256	1.1641	1.1322	1.1102	1.0899	1.0707	1.0503	1.0344	1.0236	1.0844
SL 0101-1	RF	0.0179	1.1299	1.0358	0.9612	0.8958	0.8294	0.7733	0.7216	0.6886	0.8794
	FRF	0.0149	1.0816	0.9956	0.9277	0.8677	0.8122	0.7566	0.7017	0.6723	0.8519
BIRB 0796	RF	0.0305	1.5691	1.3311	1.167	1.0297	0.9419	0.867	0.8257	0.8295	1.0701
	FRF	0.025	1.4722	1.2753	1.132	1.0112	0.9085	0.8477	0.8105	0.8169	1.0343
JNK Inhibitor VIII	RF	0.0171	1.0403	0.9667	0.8984	0.8515	0.8095	0.7649	0.7258	0.704	0.8451
	FRF	0.014	1.0046	0.9326	0.8766	0.8282	0.7845	0.741	0.6997	0.6739	0.8176
681640	RF	0.0401	1.3126	1.2982	1.2978	1.2961	1.3022	1.3173	1.3265	1.3501	1.3126
	FRF	0.0372	1.3074	1.2959	1.2947	1.2963	1.3004	1.3045	1.3135	1.3416	1.3068
Nutlin-3a	RF	0.057	1.62	1.5644	1.5465	1.53	1.5188	1.5043	1.5174	1.5027	1.538
	FRF	0.0482	1.5596	1.5159	1.4888	1.4653	1.4444	1.4282	1.4193	1.4194	1.4676
PD-173074	RF	0.0296	1.334	1.259	1.1887	1.14	1.0905	1.0454	1.0031	0.9451	1.1257
	FRF	0.024	1.284	1.2063	1.1489	1.0974	1.0486	1.0029	0.961	0.9119	1.0826
ZM-447439	RF	0.0557	1.2916	1.2784	1.2863	1.2974	1.3067	1.3279	1.36	1.3987	1.3184
	FRF	0.0583	1.2759	1.26	1.2642	1.2778	1.2975	1.324	1.3599	1.4089	1.3085
RO-3306	RF	0.0325	1.2577	1.2367	1.2154	1.1892	1.1659	1.1581	1.1488	1.1331	1.1881
	FRF	0.0281	1.243	1.2111	1.1872	1.1656	1.1445	1.1255	1.1055	1.0845	1.1584
MK-2206	RF	0.0785	1.9689	1.7278	1.5932	1.4912	1.4365	1.4328	1.4115	1.4593	1.5652
	FRF	0.0796	1.9406	1.7131	1.5751	1.4836	1.4325	1.4036	1.4055	1.4503	1.5505
PD-0332991	RF	0.0761	2.601	2.1638	1.8999	1.715	1.582	1.4882	1.4441	1.4905	1.7981
	FRF	0.0794	2.5353	2.1459	1.894	1.6994	1.5679	1.4838	1.4526	1.4822	1.7826
NVP-BEZ235	RF	0.0832	1.1133	1.0286	1.0121	0.9897	0.9926	0.9951	1.0159	1.068	1.0269
	FRF	0.0885	1.1155	1.0373	1.003	0.9856	0.9827	0.9888	1.0111	1.0685	1.0241
GDC0941	RF	0.0946	1.7499	1.6538	1.5902	1.5603	1.5251	1.509	1.5179	1.5467	1.5816
	FRF	0.099	1.764	1.6516	1.5958	1.5593	1.5351	1.52	1.5238	1.5546	1.588
AZD8055	RF	0.0734	1.0321	0.949	0.9138	0.8951	0.8889	0.8979	0.9431	1.0052	0.9406
	FRF	0.0791	1.0391	0.9537	0.9104	0.8902	0.8863	0.9012	0.9375	1.0096	0.941
PD-0325901	RF	0.0812	2.3433	2.0098	1.8288	1.7015	1.6145	1.5304	1.5044	1.5131	1.7557
	FRF	0.0769	2.2177	1.9367	1.7638	1.6408	1.5438	1.4785	1.4531	1.4715	1.6882
SB590885	RF	0.0341	1.4614	1.359	1.3116	1.2473	1.1889	1.1352	1.1127	1.0636	1.235
	FRF	0.0256	1.3478	1.2691	1.2125	1.1601	1.1085	1.0632	1.0268	0.9883	1.1471
AZD6244	RF	0.0661	2.1526	1.9101	1.7448	1.6136	1.5017	1.4053	1.3401	1.2745	1.6178
	FRF	0.0601	2.1224	1.8862	1.7305	1.5989	1.4829	1.3849	1.3119	1.2494	1.5959
AZD6482	RF	0.0598	1.927	1.5917	1.4154	1.2863	1.208	1.167	1.1855	1.2647	1.3807
	FRF	0.0608	1.9065	1.6129	1.4284	1.3027	1.2172	1.1689	1.1828	1.2614	1.3851
CCT007093	RF	0.0186	1.1204	0.9965	0.9101	0.8314	0.7611	0.7174	0.6766	0.6513	0.8331
	FRF	0.0154	1.0653	0.9586	0.8785	0.8098	0.7475	0.6955	0.6514	0.6281	0.8043
EHT 1864	RF	0.0319	1.106	1.0437	1.013	0.9898	0.986	0.9899	0.9992	1.0379	1.0207
	FRF	0.0361	1.0963	1.0257	0.9957	0.9764	0.9726	0.9781	0.9965	1.0298	1.0089

Table S2: Mean Absolute Error (MAE) between actual and predicted drug sensitivities (AUC and different IC's) for 2 methods; RF and FRF. In FRF, node cost is calculated using 8 IC regions. Here, results of all 140 drugs of GDSC database $v5$ are shown.

Drug Name	Model	AUC	IC_{10}	IC_{20}	IC_{30}	IC_{40}	IC_{50}	IC_{60}	IC_{70}	IC_{80}	Average
BMS-708163	RF	0.0293	1.5469	1.3072	1.1329	0.9999	0.8894	0.8073	0.7513	0.7346	1.0212
	FRF	0.026	1.4647	1.2511	1.1	0.9713	0.8609	0.7795	0.733	0.7283	0.9861
PF-4708671	RF	0.0292	1.1894	1.0955	1.0303	0.9893	0.9533	0.9387	0.9444	0.9531	1.0118
	FRF	0.0324	1.1888	1.0877	1.0284	0.9863	0.9572	0.9354	0.9332	0.9525	1.0087
JNJ-26854165	RF	0.0497	0.9948	0.9099	0.8831	0.888	0.9249	0.9739	1.0338	1.1382	0.9683
	FRF	0.0542	1.0047	0.9112	0.879	0.8797	0.9081	0.9501	1.0172	1.1193	0.9587
TW 37	RF	0.0997	1.1688	1.0726	1.0199	1.0149	0.9969	1.0216	1.0692	1.1546	1.0648
	FRF	0.1119	1.1604	1.0587	1.0154	1.0005	1.0044	1.0245	1.0704	1.1612	1.0619
CCT018159	RF	0.0444	0.9253	0.9811	1.0433	1.0947	1.1444	1.198	1.269	1.3503	1.1258
	FRF	0.0455	0.8995	0.9516	1.0032	1.0567	1.1122	1.1703	1.239	1.3314	1.0955
AG-014699	RF	0.032	1.2495	1.2112	1.1764	1.1557	1.1313	1.113	1.1076	1.0955	1.155
	FRF	0.0295	1.2243	1.179	1.1516	1.1296	1.1092	1.094	1.0816	1.0694	1.1298

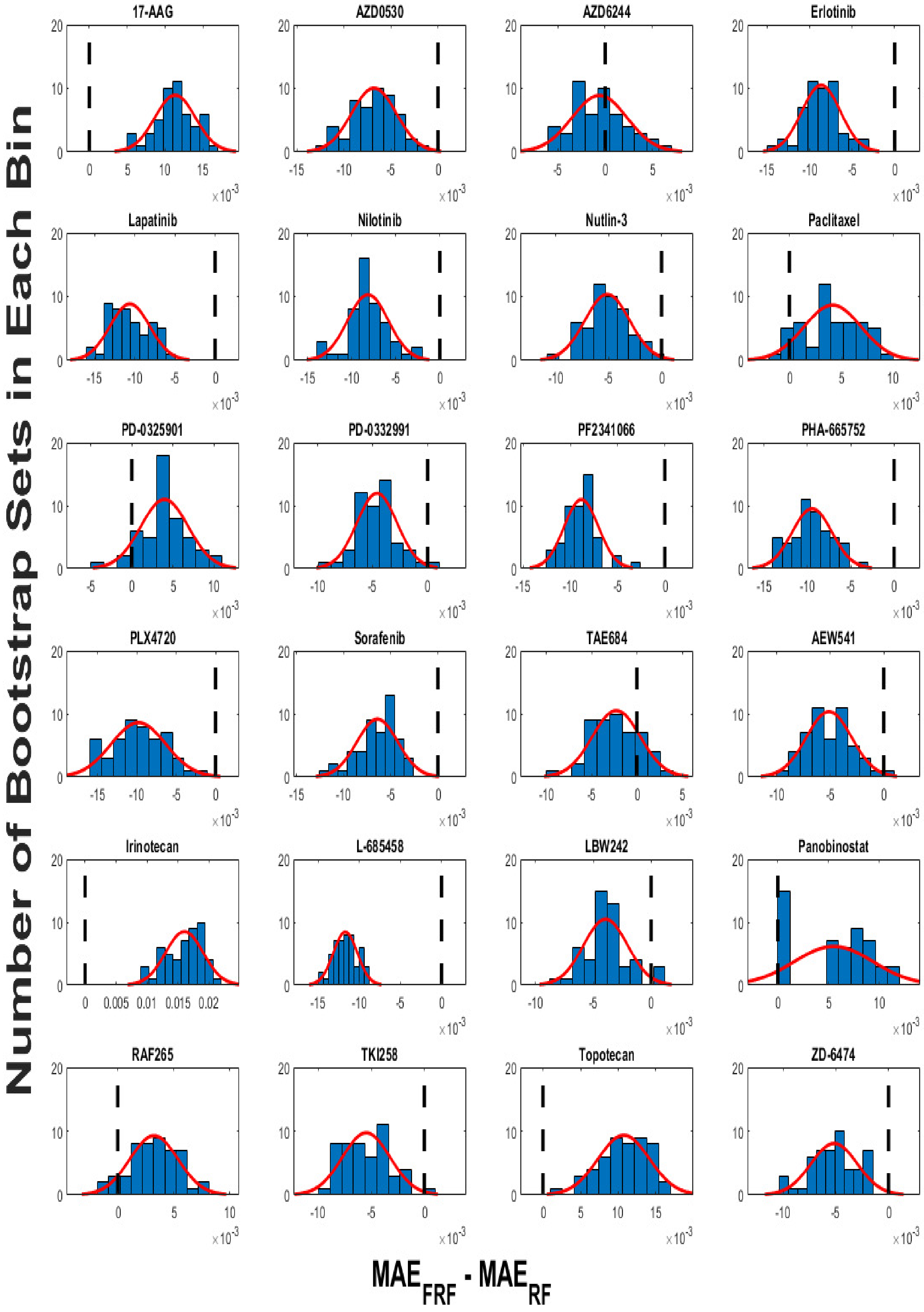

Figure S2: Distributions of MAE differences between FRF and RF predictions for the 50 bootstrap set show the robustness of FRF models.

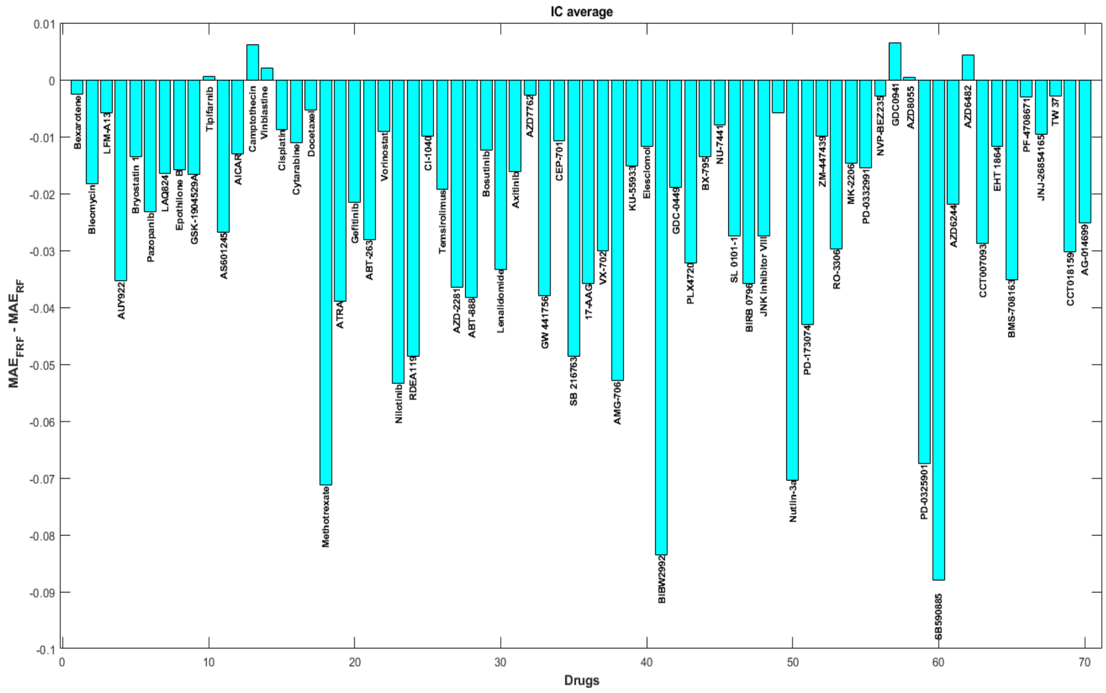

Figure S3: Difference between MAE of FRF and MAE of RF for Average IC values for 70 drugs of GDSC (rest 70 in main manuscript).
